# An RNA-targeting CRISPR–Cas13d system alleviates disease-related phenotypes in Huntington’s disease models

**DOI:** 10.1038/s41593-022-01207-1

**Published:** 2022-12-12

**Authors:** Kathryn H. Morelli, Qian Wu, Maya L. Gosztyla, Hongshuai Liu, Minmin Yao, Chuangchuang Zhang, Jiaxu Chen, Ryan J. Marina, Kari Lee, Krysten L. Jones, Megan Y. Huang, Allison Li, Charlene Smith-Geater, Leslie M. Thompson, Wenzhen Duan, Gene W. Yeo

**Affiliations:** 1 Department of Cellular and Molecular Medicine, University of California San Diego, La Jolla, CA USA; 2 Division of Neurobiology, Department of Psychiatry and Behavioral Sciences, Johns Hopkins University School of Medicine, Baltimore, MD USA; 3 School of Traditional Chinese Medicine, Beijing University of Chinese Medicine, Beijing, China; 4 Department of Psychiatry and Human Behavior, University of California Irvine, Irvine, CA USA; 5 Department of Neurobiology and Behavior, University of California Irvine, Irvine, CA USA; 6 The Solomon H Snyder Department of Neuroscience, Johns Hopkins University School of Medicine, Baltimore, MD USA; 7 Program in Cellular and Molecular Medicine, Johns Hopkins University School of Medicine, Baltimore, MD USA; 8 Institute for Genomic Medicine, University of California San Diego, La Jolla, CA USA; 9 Stem Cell Program, University of California San Diego, La Jolla, CA USA

**Keywords:** Huntington's disease, Gene therapy, Molecular engineering

## Abstract

Huntington’s disease (HD) is a fatal, dominantly inherited neurodegenerative disorder caused by CAG trinucleotide expansion in exon 1 of the huntingtin (*HTT*) gene. Since the reduction of pathogenic mutant *HTT* messenger RNA is therapeutic, we developed a mutant allele-sensitive CAG^EX^ RNA-targeting CRISPR–Cas13d system (Cas13d–CAG^EX^) that eliminates toxic CAG^EX^ RNA in fibroblasts derived from patients with HD and induced pluripotent stem cell-derived neurons. We show that intrastriatal delivery of Cas13d–CAG^EX^ via an adeno-associated viral vector selectively reduces mutant *HTT* mRNA and protein levels in the striatum of heterozygous zQ175 mice, a model of HD. This also led to improved motor coordination, attenuated striatal atrophy and reduction of mutant HTT protein aggregates. These phenotypic improvements lasted for at least eight months without adverse effects and with minimal off-target transcriptomic effects. Taken together, we demonstrate proof of principle of an RNA-targeting CRISPR–Cas13d system as a therapeutic approach for HD, a strategy with implications for the treatment of other dominantly inherited disorders.

## Abstract (web summary)

Leveraging RNA-targeting CRISPR–Cas13d technology, Morelli et al. engineered a novel therapeutic strategy that safely and effectively eliminates toxic expanded huntingtin RNA in multiple models of Huntington’s disease.

## Main

HD is an autosomal dominant neurodegenerative disorder caused by a CAG short tandem repeat (STR) expansion in exon 1 of the huntingtin (*HTT*) gene^1^. This trinucleotide sequence codes for the amino acid glutamine (Q), placing HD in a broader class of neurological disorders known as polyglutamine (polyQ) diseases. Motor symptoms can manifest from childhood to old age, with onset inversely correlated with CAG repeat length in mutant *HTT*, with the longer the CAG repeat, the earlier symptoms arise^2,3^. The therapies currently available to patients with HD offer only moderate symptomatic relief and affected individuals typically die 15–20 years post-diagnosis due to complications^2,4^. Therefore, a major focus of HD therapeutic development has shifted toward targeting the root of the disease through depletion of mutant *HTT*^5,6^. Besides the toxicity of the mutated HTT protein, an increasing body of evidence indicates that mutant HTT mRNA also contributes to disease pathogenesis^7,8^. Consequently, strategies to suppress both HTT transcripts and protein levels would be most beneficial as a treatment. RNA interference (RNAi) and antisense oligonucleotide (ASO) strategies have shown preclinical efficacy and are being tested in clinical trials^6^. However, most of these approaches do not precisely differentiate mutant HTT from the normal allele^9^.

Most patients with HD are heterozygous for the CAG expansion and rely on their normal *HTT* allele to play important roles during brain development as well as in adult central nervous system (CNS) function^10^. In the adult brain, HTT helps regulate intracellular vesicle trafficking^11–13^, transcriptional regulation^14,15^ and synaptic connectivity^16–18^. While partial reduction of normal HTT levels is tolerable in multiple preclinical animal models of HD^19,20^, long-term ramifications of reductions in humans are unclear given its involvement in a myriad of biological functions^21^. Sustained reduction of normal HTT levels may even exacerbate HD pathogenesis^10,22^. Indeed, the recent termination of the phase III clinical trial (NCT03761849) of a non-allele-selective HTT-lowering ASO developed by Roche in patients with HD underscores the urgency to develop an allele-selective strategy that can selectively and effectively suppress mutant HTT mRNA expression^23–26^.

The RNA-guided, RNA-targeting subtype of VI-D CRISPR–Cas, Cas13d, has been recently identified as an efficient and specific RNA-targeting approach that can be applied in mammalian cells^27^. Cas13d, with a size of only approximately 930 amino acids, can be packaged and delivered with its single-guide RNAs (sgRNAs) to target cells via a single adeno-associated virus (AAV) capsid. Cas13d possesses dual RNase activities and is capable of processing CRISPR arrays and cleaving target RNAs in a protospacer flanking sequence-independent manner^27–29^. In cell-based screenings and side-by-side comparisons to short-hairpin RNA (shRNA), nuclear localized sequence-fused Cas13d showed a strong ability to cleave target RNA with high efficiency (approximately 96% knockdown by Cas13d compared to approximately 65% by shRNA) and high specificity with minimal off-target effects in mammalian cell culture^28^. These attributes indicate that CRISPR–Cas13d is a promising platform for RNA targeting in clinical applications such as selective mHTT depletion. Unlike gene therapies engineered from DNA-targeting CRISPR systems—which can cause irreversible nonspecific and unintended genetic modifications in the patient’s genome, that can be inherited in subsequent generations—targeting RNA appears a safer, viable alternative.

In this study, we tested a CRISPR–Cas13d-based gene therapy approach that silences mutant toxic CAG-expanded (CAG^EX^) HTT RNA in both human and mouse models of HD. Our CAG^EX^-targeting Cas13d system (Cas13d–CAG^EX^) selectively reduces CAG^EX^ HTT RNA in neurons derived from patients with HD with CAG expansions ranging from 66 to 109 STRs. AAV-mediated delivery of Cas13d–CAG^EX^ to the striatum of premanifest zQ175/+ HD mice resulted in allele-selective suppression of mutant HTT mRNA and protein aggregates while maintaining normal HTT mRNA and protein levels, significantly improved motor function and attenuated striatal atrophy. Our data provide the first evidence, to our knowledge, that CRISPR–Cas13d-mediated elimination of mutant mRNA and protein is a promising allele-selective therapeutic strategy.

## Results

### Development of a Cas13d system that targets CAG^EX^

**Fig. 1:**
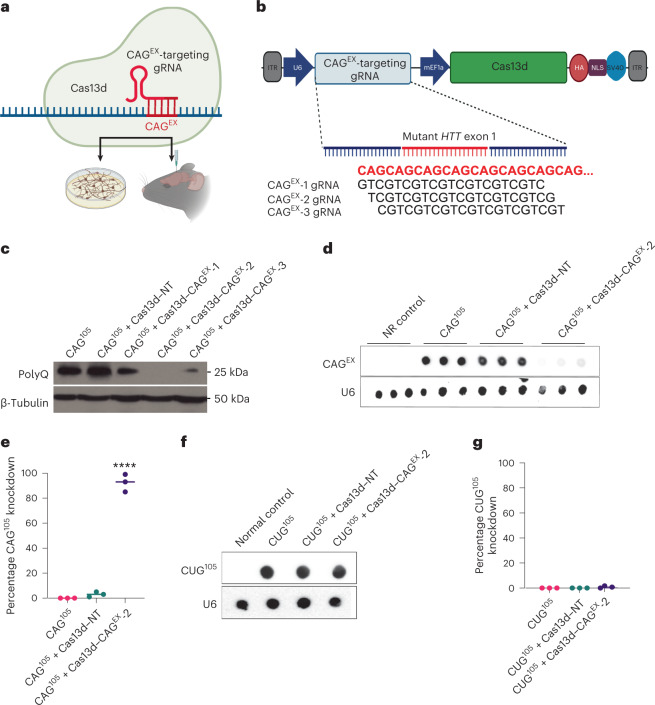
Development of an RNA-targeting, Cas13d-based gene therapy approach for HD. **a**, Treatment scheme of our single gene therapy that expresses Cas13d and a gRNA designed to eliminate CAG-expanded HTT *HTT*RNA in both human striatal neuronal cultures derived from patient iPSCs and in the striatum of an established mouse model of HD, zQ175/+. **b**, Diagram of a series of CAG-expanded, RNA-targeting vectors that consists of (1) Cas13d tagged with an HA epitope and (2) one of three U6 promoter-driven Rfx CRISPR–Cas13d gRNAs (denoted as CAG^EX^ gRNA 1–3). **c**, Western blot analysis of polyQ protein from protein lysates isolated from HEK293 cells transfected with a CAG^105^ repeat plasmid and each candidate Cas13d vector. **d**,**e**, RNA dot blot analysis (**d**) and quantification (**e**) of CAG-expanded RNA within HEK293 cells transfected with a CAG^105^ repeat plasmid along with a nontargeting control (NT) or CAG^EX^-2 vector (one-way ANOVA, Tukey post hoc test, ****P < 0.0001; *n* = 1 technical replicates, *n* = 3 biological replicates). **f**,**g**, RNA dot blot analysis (**f**) and quantification (**g**) of CUG-expanded RNA within HEK293 cells transfected with a CUG^105^ repeat plasmid along with a NT or CAG^EX^-2 vector (one-way ANOVA, Tukey post hoc test, *n* = 3 technical replicates, *n* = 3 biological replicates). Source data

Although the etiology of HD is complex, many proposed mechanisms arise from the transcription and subsequent translation of CAG^EX^ HTT, ultimately causing disease through a toxic gain-of-function mechanism. Therefore, therapeutic approaches that suppress mutant HTT at the RNA level are actively being pursued. We recently pioneered the repurposing of the Cas9 system to target and eliminate toxic repeat RNAs in vitro^30^ and in vivo^31^ delivered by a two-vector AAV system, demonstrating that RNA-targeting CRISPR approaches are effective with RNA repeat expansions. In this study, due to the smaller size and natural RNA-targeting capacity of *Ruminococcus flavefaciens* XPD3002 (Rfx) CRISPR–Cas13d^28^, we developed a CAG^EX^ RNA-targeting Cas13d (Cas13d–CAG^EX^) system that we packaged into a single vector in both lentiviral and AAV delivery vehicles to evaluate its therapeutic potential in multiple established preclinical models of HD. These included fibroblasts from patients with HD, differentiated neurons with striatal characteristics from a panel of induced pluripotent stem cell (iPSC) lines from patients with HD and a full-length mutant *HTT* knock-in mouse model expressing a human mutant exon-1 with the expanded CAG repeat (approximately 220 repeats) within the native mouse huntingtin gene, zQ175/+ (Fig. 1a).

First, to optimize knockdown of CAG^EX^ HTT RNA by Cas13d, we engineered three distinct RNA-targeting vectors that consist of (1) Cas13d tagged with a human influenza hemagglutinin (HA) epitope and (2) one of three U6 promoter-driven Cas13d gRNAs (denoted as CAG^EX^ gRNA 1–3). All three gRNAs are complementary to the CAG^EX^ RNA sequence with each guide targeting a different codon within the repeat expansion: CAG^EX^ gRNA-1 (GTC), CAG^EX^ gRNA-2 (TCG) and CAG^EX^ gRNA-3 (CGT) (Fig. 1b). For an initial assessment of the capability of each CAG^EX^ gRNA to knockdown CAG^EX^ RNA, HEK293 cells were cotransfected with a repeat expansion plasmid with 105 CAG STRs along with a Cas13d-containing vector with a nontargeting gRNA designed to target a sequence from the λ bacteriophage (Cas13d–NT) or one of the three CAG^EX^-targeting guides as described previously^30^. Since aggregation of toxic polyQ protein translated from CAG^EX^ RNA is well documented as one of the primary hallmarks of HD neuropathology, we determined if and to what extent Cas13d in conjunction with each CAG^EX^-targeting gRNA can eliminate polyQ protein in live human cells. We observed that polyQ protein produced by this plasmid was dramatically reduced in cells cotransfected with Cas13d and CAG^EX^ gRNA-2 compared to cells only transfected with the CAG^105^-expressing plasmid via western blot with a polyQ-specific antibody (MAB1574)^30^ (Fig. 1c). RNA blots performed with RNA isolated from cells cotransfected with our CAG repeat expression plasmid and Cas13d with the CAG^EX^-2 gRNA also showed a significant (85.4%) reduction of CAG^EX^ RNA compared with cells transfected with our nontargeting Cas13d vector (one-way analysis of variance (ANOVA), ****P < 0.001) (Fig. 1d, quantified in Fig. 1e). Importantly, expression studies using CUG expansion (CUG^EX^) RNA-expressing plasmids and a semiquantitative RNA dot blot were performed to determine if Cas13d with the CAG^EX^-2 gRNA specifically eliminates RNA transcripts that contain CAG expansions without degrading similar GC-rich transcripts such as those with CUG expansions. Excitingly, we observed that Cas13d with the CAG^EX^-2 gRNA specifically targets and degrades CAG^EX^ transcripts while leaving CUG^EX^ transcripts intact (Fig. 1f, quantified in Fig. 1g). Taken together, these data confirm that our Cas13d system, which includes the CAG^EX^-2 gRNA, now referred to as ‘Cas13d–CAG^EX^’, can effectively eliminate CAG^EX^ RNA and subsequent polyQ protein in human cells possibly without targeting other trinucleotide repetitive elements such as CUG^EX^.

### Cas13d–CAG^EX^ reduces m*HTT* in cells from patients with HD

**Fig. 2:**
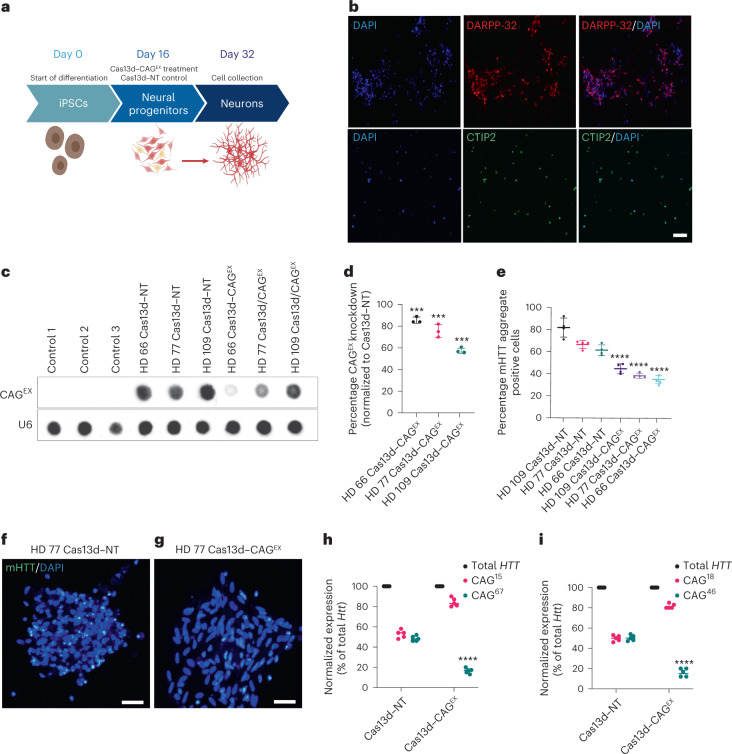
Cas13d–CAG^EX^ reduces m*HTT* mRNA and protein in cells derived from patients with HD. **a**, Schematic of the differentiation protocol used for iPSC-derived neurons treated with a lentiviral system expressing Cas13d–CAG^EX^. **b**, Representative immunofluorescence images of cells at day 32 in control neuronal cells, showing that the cells are positive for striatal markers, DARPP-32 (top) and CTIP2 (bottom). Scale bar, 20 μm. *n* = 1 technical replicate. **c**,**d**, RNA dot blot analysis (**c**) and quantification (**d**) of CAG-expanded RNA within MSN cells transduced with Cas13d–NT- or Cas13d–CAG^EX^-expressing lentiviral vectors (one-way ANOVA, Tukey post hoc test, ***P < 0.001). HD 66 Cas13d–CAG^EX^ *P* = 0.00037, HD 77 Cas13d–CAG^EX^ *P* = 0.00054, HD 109 Cas13d–CAG^EX^ *P* = 0.00067; *n* = 3 technical replicates, *n* = 3 biological replicates. Data are presented as mean values ± s.e.m. **e**–**g**, Quantification of mHTT aggregates within MSN cells transduced with Cas13d–NT- or Cas13d–CAG^EX^-expressing lentiviral vectors (**e**) (one-way ANOVA, Tukey post hoc test, ****P < 0.0001, *n* = 3 technical replicates, *n* = 4 differentiations, 200 cells per experimental group). Representative images of the analysis are in **f** and **g** (scale bar, 20 μm). Data are presented as mean values ± s.e.m. **h**,**i**, Quantification of mutant and WT HTT RNA via allele-specific RT-qPCR in GM04723 (**h**) and GM02151 (**i**) fibroblasts (two-way ANOVA, Tukey post hoc test, ****P < 0.0001, *n* = 5 biological replicates). DAPI, 4,6-diamidino-2-phenylindole. Source data

**Extended Data Fig. 1:**
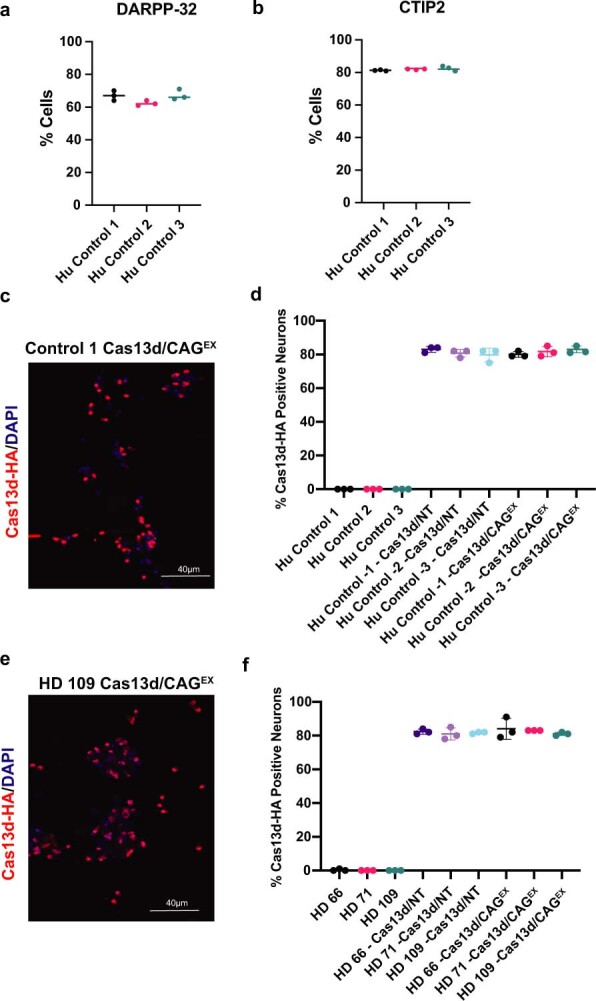
Cas13d distribution in iPSC-derived neurons with striatal characteristics. **a**, **b**. Quantification of DARPP-32-, and CTIP2- positive cells within day 32 control iPSC-derived neurons with striatal characteristics (3 differentiations per cell lines, 1500 cells per experimental group). **c-f**. Image and quantification of Cas13d-HA distribution in Control and HD Day 32 neuronal cultures (n = 3 differentiations cell line per experimental group) Data are presented as mean values ± SEM. Scale bar = 40 µm. Source data

To evaluate Cas13d–CAG^EX^ in a human preclinical model, a panel of neuronal cultures enriched for striatal characteristics was generated from three previously validated iPSC lines derived from individual patients with HD. These independent lines contained repeats in the *HTT* locus ranging from 66 to 109 CAGs (referred to as HD 66, 77 and 109) and were compared to 3 non-isogenic, neurotypical iPSC lines isolated from three different individuals with <25 CAG repeats in exon 1 of *HTT*. Although not age- or sex-matched to our lines from patients with HD, the control samples (controls 1–3) used to evaluate the effects of different lengths of CAG expansions on human neurons have been characterized in previous publications^32–34^ and checked for aberrant genomic alterations via karyotype and copy number variation (CNV) arrays to avoid line-specific confounds in downstream analyses. Each HD and control neuronal culture was transduced with a constitutive, lentiviral system supplying either Cas13d–CAG^EX^ or nontargeting Cas13d–NT at 16 days post-differentiation, when most of each culture consisted of neuroprogenitor cells (Fig. 2a). At day 32 post-differentiation, both HD and control cultures consisted of neurons positive for striatal markers, dopamine- and cAMP-regulated neuronal phosphoprotein (DARPP-32) and COUP-TF1-interacting protein 2 (CTIP2) (Fig. 2b, quantified in Extended Data Fig. 1a,b). A high percentage of DARPP-32 (65.5%) and CTIP2 (82.3%) was observed indicating that our human cell cultures consisted of neurons enriched for striatal characteristics. Widespread transduction was confirmed with Cas13d-HA immunofluorescence with 78% or more (average 83.4%) of neurons showing Cas13d expression (Extended Data Fig. 1c–f). RNA slot blot hybridization detected that CAG^EX^ RNA was significantly reduced by Cas13d–CAG^EX^ with an 84.1% reduction in HD 66, a 79.8% reduction in HD 77 and a 56.2% reduction in HD 109 (Fig. 2c, quantified in Fig. 2d). Since protein aggregates are pathological hallmarks of HD, we determined the effect of Cas13d–CAG^EX^ on mutant HTT aggregation, immunolabeled by the EM48 antibody. Cas13d–CAG^EX^-treated HD lines had significantly reduced mutant HTT aggregates compared with those treated with Cas13d–NT (Fig. 2e, representative images in Fig. 2f,g), indicating that Cas13d–CAG^EX^ can selectively suppress mutant HTT aggregates.

To evaluate the allele-selectivity of Cas13d–CAG^EX^ on shorter repeats more common in adult-onset patients, we utilized two human fibroblast lines from patients with HD with (67/15) CAG repeats (GM04723) and (46/18) CAG repeats (GM02151). Allele-specific quantitative PCR with reverse transcription (RT–qPCR) using patient-specific single-nucleotide polymorphism (SNP)-based primers showed a significant and specific knockdown of mutant HTT RNA in each patient fibroblast line treated with Cas13d–CAG^EX^-expressing lentiviral vectors compared to those transfected with Cas13d–NT (Fig. 2h,i). These data support the broad utility of Cas13d–CAG^EX^ for patients with adult- or juvenile-onset HD-relevant CAG repeat lengths in mutant *HTT*.

### Cas13d–CAG^EX^ reduces specific molecular biomarkers in patients with HD

**Fig. 3:**
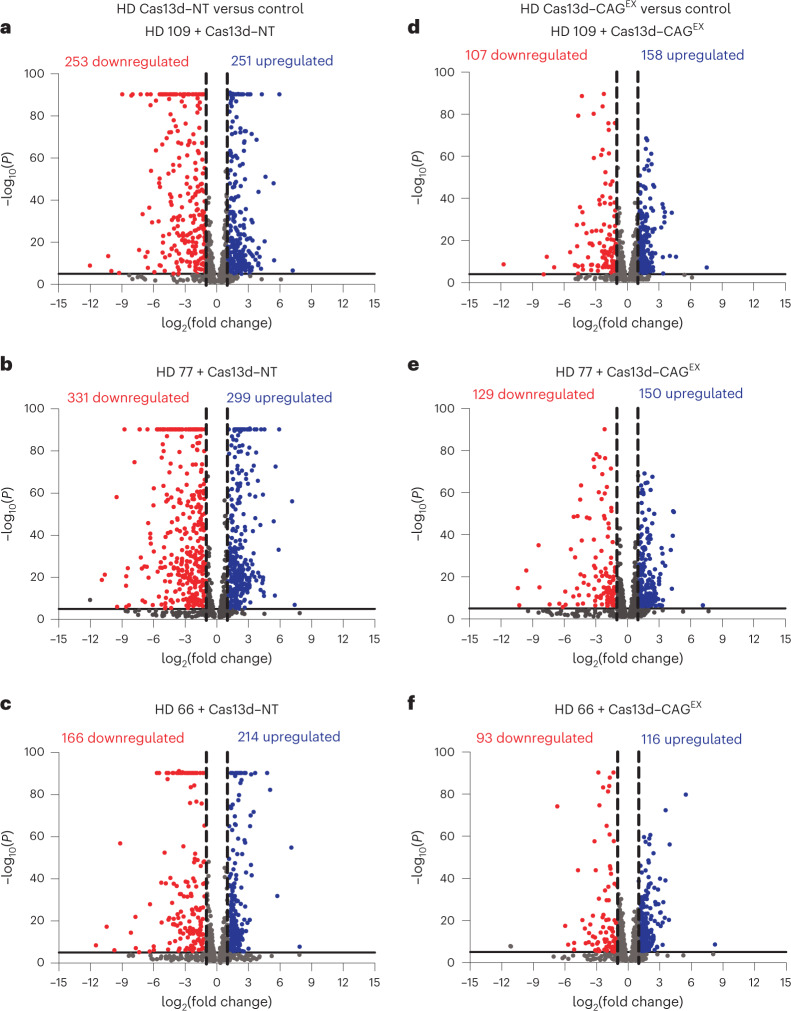
Cas13d–CAG^EX^ partially reverses the molecular phenotypes of HD in patient iPSC-derived neurons. **a**–**f**, Scatter plots of a common list of 988 upregulated and downregulated DEGs within HD 109, HD 77 and HD 66 treated with either Cas13d–NT (HD 109 (**a**), HD 77 (**b**), and HD 66 (**c**)) or Cas13d–CAG^EX^ (HD 109 (**d**), HD 77 (**e**), and HD 66 (**f**)) lentivirus vector compared to controls 1–3 referred to as ‘control’. The most significant HD-associated DEGs defined by a twofold change from control and a FDR-adjusted *P* < 0.00001 are distinguished from total HD-associated DEGs with a broken vertical black line on the *x* axis (log_2_ fold change) and a solid gray line on the *y* axis (−log_10_(*P*)), showing partial reversal of HD-mediated changes in the human transcriptome defined in **a**–**c** by Cas13d–CAG^EX^ in **d**–**f**.

**Extended Data Fig. 2:**
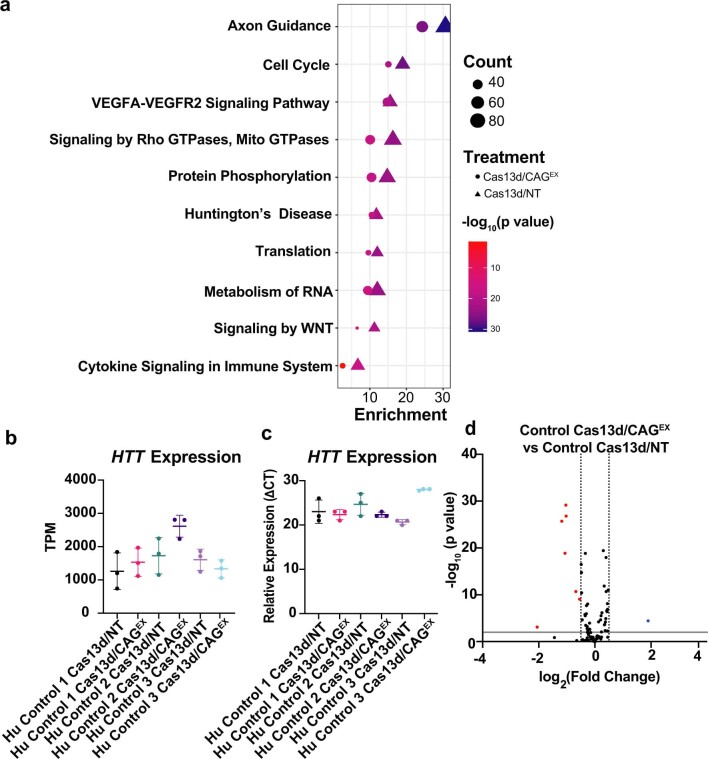
Allele-specificity and safety of Cas13d/CAG^EX^ in human iPSC-derived neurons with striatal characteristics. **a**. GO analysis of 988 differentially expressed genes that distinguish our HD MSN lines from controls as well as HD DEGs reversed by Cas13d/CAG^EX^. Significant GO terms were determined by Fisher’s exact test after FDR correction at p < 0.05 and sorted by fold enrichment. **b**, **c**. Quantification of total *HTT* transcript by TPM and quantitative PCR within control neuronal cultures with Cas13d/NT or Cas13d/CAG^EX^ (n = 3 per experimental group) Data are presented as mean values ± SEM. **d**. Scatter plot of CAG-expanded transcripts in the human transcriptome within control neuronal lines treated with either Cas13d/NT or Cas13d/CAG^EX^ (FDR-adjusted p value < 0.01). Source data

Transcriptome-wide RNA sequencing (RNA-seq) analysis utilizing the DESeq2 package^35^ identified 988 differentially expressed genes (DEGs) (false discovery rate (FDR)-adjusted *P* < 0.0001) that distinguish our HD neuronal lines from controls (Fig. 3a–c and Supplementary Table 1). Gene ontology (GO) biological process analyses revealed that these DEGs were enriched for terms associated with neurodegenerative disorders including ‘Rho GTPases’, ‘translation’ and the ‘Vascular endothelial growth factor A–vascular endothelial growth factor receptor 2 signaling pathway’. The term ‘Huntington disease’ was also enriched and included genes associated with known HD-mediated pathological pathways such as cytosolic and mitochondrial calcium overload, endoplasmic reticulum stress through proteasomal dysfunction and impaired autophagy function^36–44^ (Fisher’s exact test with FDR-adjusted *P* < 0.05; Extended Data Fig. 2a and Supplementary Table 2). Therefore, we opine that these gene expression changes are a useful measure of the efficacy of our Cas13d-based approach. In comparing individual HD neuronal cultures treated with Cas13d–CAG^EX^ or Cas13d–NT to all 3 control lines treated with Cas13d–NT, we determined that 61.2% (605 out of 988 DEGs), 77.1% (762 out of 988 DEGs) and 57.5% (568 out of 988 DEGs) of HD DEG signatures were partially reversed in the HD 109, HD 77 and HD 66 lines, respectively, treated with Cas13d–CAG^EX^, with evident reversal defined as a fold change of 50% or more in the expression of each DEG toward wild-type (WT) levels satisfying an FDR-adjusted *P* < 0.01 (Supplementary Table 1). Importantly, reversed DEGs were enriched for each HD-associated pathological pathway mentioned above, suggesting that our gene therapy can alleviate previously reported downstream effects of m*HTT* (Extended Data Fig. 2a and Supplementary Table 2). We also made the critical observation that 48% (HD 109), 56% (HD 77) and 45% (HD 66) of the most robust HD DEGs (twofold change, *P* < 0.00001) were partially reversed (Fig. 3d–f). In addition to the common 988 DEGs, excitingly, we also determined that patient-specific DEGs (FDR-adjusted *P* < 0.0001) were also partially reversed by 76.7% (4,582 out 5,973) in HD 109 and 81.3% (4,870 out of 5,991) in HD 77 and to a lesser degree (36.2%; 1,830 out of 5,055) in HD 66 (which harbored only approximately 80% of the extent of changes in HD 77 and HD 109) by Cas13d–CAG^EX^ treatment (Supplementary Table 3). Our results demonstrate that our gene therapy approach reduces the most prominent disease markers common in both groups and individual patients.

Importantly, neither quantification of HTT mRNA by transcript expression levels (transcripts per million) nor qPCR could detect a significant change in total HTT mRNA levels in any of the 3 control lines treated with Cas13d–CAG^EX^ compared to Cas13d–NT or left untreated (one-way ANOVA, Tukey’s post hoc test, *P* < 0.05) (Extended Data Fig. 2b,c). To assess the potential off-target effects of Cas13d–CAG^EX^ on other transcripts with non-disease-associated CAG repeats, we next examined the expression levels of protein-coding transcripts with five or more CAG, AGC or GCA tandem repeats, which could be inadvertently targeted by the CAG^EX^ gRNA. Of 92 transcripts with five or more repeats, seven statistically significant (twofold change, FDR-adjusted *P* < 0.01) DEGs were detected (*ZNF853*, *MN1*, *IRS1*, *MSH4*, *VASH2*, *LMX1A*, *ST6GALNAC5*; Extended Data Fig. 2d and Supplementary Table 4). Considering that most of these genes are involved in neurodevelopment, including transcriptional regulation of developmental genes (*ZNF8585*, *MN1*, *LMX1A*) and axon development (*VASH2*), it is uncertain how their dysregulation will affect the adult brain. However, none of these DEGs are associated with adult-onset neurological disorders. These data suggest that other CAG-containing genes may be minimally affected by our Cas13d-based gene therapy approach.

### Therapeutic efficacy and safety of Cas13d–CAG^EX^ in vivo

**Fig. 4:**
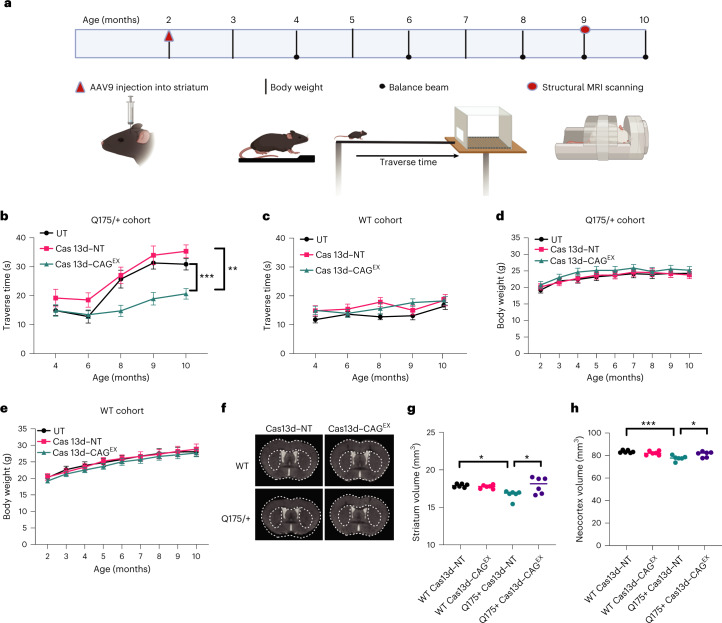
Therapeutic efficacy of Cas13d–CAG^EX^ in a full-length knock-in mouse model of HD. **a**, Timeline of experimental design and outcome measures. **b**–**e**, Motor function and body weight. Mice were tested on a 5-mm balance beam and time crossing the beam (traverse time) was recorded at the indicated ages in zQ175/+ HD (**b**) or WT mice (**c**) with the indicated treatments. ***P = 0.0004, **P = 0.0057, two-way ANOVA with Bonferroni post hoc tests. Body weight in zQ175/+ HD mice (**d**) or WT mice (**e**) with the indicated treatments (*n* = 10, 5 males and 5 females per group). Data are presented as mean values ± s.e.m. **f**, Representative MRI images of the zQ175/+ HD and WT mice injected with the indicated AAVs. **g**,**h**, Striatal (**g**) and neocortex (**h**) volume was quantified from 3D structural MRI in the indicated groups at 9 months of age (7 months after AAV injections). *P < 0.05, ***P < 0.001, one-way ANOVA with Tukey analysis. *n* = 6 mice per group, 3 females and 3 males per group, 9 months of age. **g**, *P* (Q175 + Cas13d–NT versus Q175 + Cas13d–CAG^EX^) = 0.0196; *P*(Q175 + Cas13d–NT versus WT + Cas13d–NT) = 0.0241. **h**, *P*(Q175 + Cas13d–NT versus Q175 + Cas13d–CAG^EX^) = 0.0451; *P*(Q175 + Cas13d–NT versus WT + Cas13d–NT) = 0.0005. Source data

**Extended Data Fig. 3:**
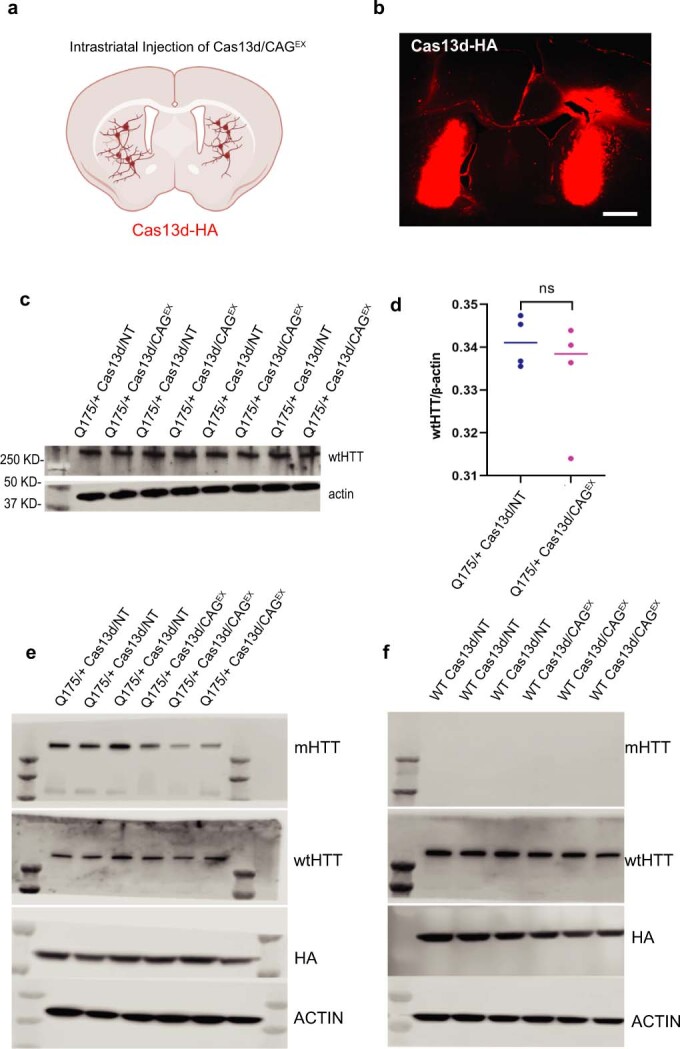
Allele-specificity and safety of Cas13d/CAG^EX^ in a full-length mHTT knock-in mouse model. **a, b**. Detection of by Cas13d-HA via HA immunostaining (red) 3 weeks post-intrastriatal injection of AAV9-Cas13d/CAG^EX^. Scale bar = 900 µm (b). **c, d**. Western blot analysis of wild type HTT (wtHTT, antibody MAB 2166 antibody). There are no significant differences between two groups by a one-sided Student’s *t*-test (n = 4 mice/group, 2 female and 2 male per group, 11 weeks of age). **e, f**. Full images of western blots shown in Fig. 5e, f. Source data

**Extended Data Fig. 4:**
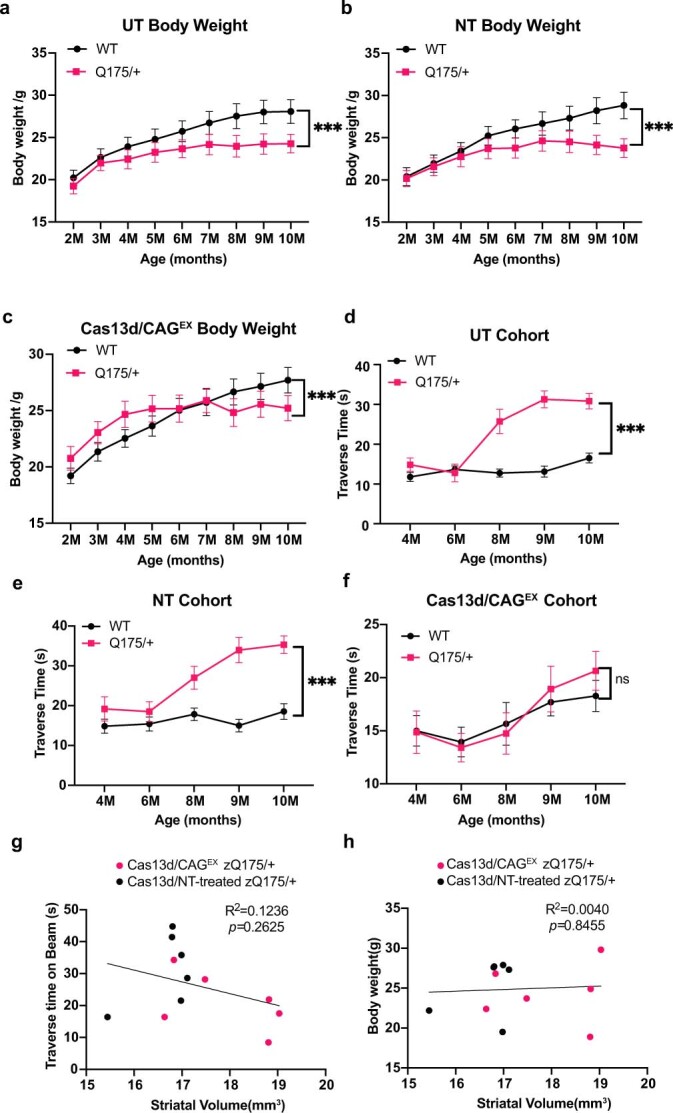
Body weight, motor function, and the correlation with striatal volume in a full-length mHTT knock-in mouse model of HD. **a-c**. a-c. Body weight in zQ175/+ HD mice or WT mice with indicated treatments. ***p < 0.001, two-way ANOVA with Bonferroni post hoc tests for Fig. 4b; ***p < 0.001, Mixed-effects model with Bonferroni post hoc tests for Fig. 4a, c. n = 10, 5 male and 5 female/group. Data are presented as mean values ± SEM. **d-f**. Mice were tested on a 5 mm balance beam and time crossing the beam (Traverse time) was recorded at indicated ages in zQ175 HD mice or WT mice with indicated treatments. ***p < 0.001, two-way ANOVA with Bonferroni post hoc tests. n = 10, 5 male and 5 female/group Data are presented as mean values ± SEM. **g, h**. The correlation analysis (Goodness of Fitness Test) between striatal volume and motor function (g) or body weight (h) in the zQ175/+ mice treated with Cas13d/CAG NT or CAG^EX^. No significant correlation was detected between striatal volume and motor function (p = 0.1236) or body weight (p = 0.8455) in the zQ175 treated with either Cas13d/CAG NT or CAG^EX^. Source data

To determine if our approach can prevent or halt the progression of neurodegenerative phenotypes in vivo in zQ175/+ mice, we packaged Cas13d–CAG^EX^ or Cas13d–NT into single-stranded AAV serotype 9 (AAV9) viral vectors and conducted bilateral intrastriatal injection of Cas13d–CAG^EX^ or Cas13d–NT to an equal number of two-month-old zQ175/+ HD mice and age-matched WT littermate controls. At three weeks postinjection, we observed successful expression of both Cas13d–HA fusion constructs in mouse striatum, indicated by red fluorescent signals of HA protein with an anti-HA antibody (Extended Data Fig. 3a,b). Motor function and body weight were monitored longitudinally over eight months postinjection as illustrated (Fig. 4a) and mHTT aggregates, HTT mRNA and protein levels were determined at the end of the study. We evaluated the effect of treatment on motor function on a balance beam. While zQ175/+ HD mice exhibited clear motor deficits at eight months of age and after; those treated with Cas13d–CAG^EX^ showed significantly improvements in motor performance, indicated by shorter traverse times, compared with zQ175/+ mice treated with Cas13d–NT (*P* < 0.001) or left untreated (*P* < 0.01) (Fig. 4b and Extended Data Fig. 4d–f). Importantly, Cas13d–CAG^EX^ had no effect on motor function in WT mice (Fig. 4c) and did not alter body weight in zQ175/+ (Fig. 4d) or WT mice (Fig. 4e), implying that Cas13d–CAG^EX^ does not produce gross adverse effects in mice.

Next, we examined whether Cas13d–CAG^EX^-mediated mHTT silencing could improve neuropathology in zQ175/+ mice. Using human-translatable high-resolution structural magnetic resonance imaging (MRI), we delineated regional brain volumes accurately using an automatic segmentation, large deformation diffeomorphic metric mapping (LDDMM) including the striatum and neocortex^45–47^. We performed MRI scans of nine-month-old zQ175/+ mice, when these HD mice display striatal atrophy as we reported previously^47^. Our behavioral results indicate that Cas13d–NT has no significant effect compared to the untreated control mice; thus, we focused on four groups in the MRI study: WT or HD mice injected with Cas13d–NT or Cas13d–CAG^EX^. We demonstrate that zQ175/+ mice injected with Cas13d–NT exhibit significantly reduced striatal volume and neocortex volume compared to WT mice with the same injection, while Cas13d–CAG^EX^-injected zQ175/+ mice have preserved striatal volume and neocortex volume compared to HD mice injected with Cas13d–NT (by group mean analysis; Fig. 4f–h and Supplementary Table 5). In addition, we performed correlation analysis between striatal volume and traverse time or body weight in zQ175/+ mice treated with either Cas13d–NT or Cas13d–CAG^EX^ and found that there was no significant correlation between striatal volume and traverse time in zQ175/+ mice (*P* = 0.2625; Extended Data Fig. 4g) or body weight (*P* = 0.3755; Extended Data Fig. 4h). We note that the brain volume data comparison is between two groups of HD mice treated with either Cas13d–CAG^EX^ or Cas13d–NT. By considering the brain volumetric measurement of individual zQ175/+ mice, our results suggest that the individual variability in striatal volume of zQ175/+ mice in each treatment group is mainly due to natural variation rather than different responders to the treatment.

### Selective m*HTT* knockdown by Cas13d–CAG^EX^ in zQ175/+ mice

**Fig. 5:**
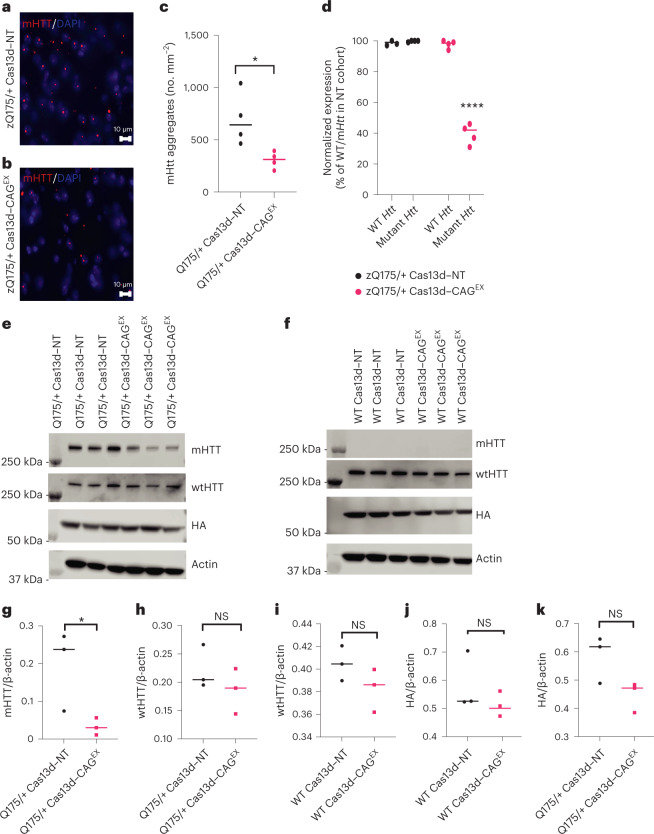
Allele-specific knockdown of mutant HTT protein and mRNA by Cas13d–CAG^EX^ in a full-length knock-in mouse model of HD. **a**,**b**, Representative mHTT aggregates detected by immunostaining with EM48 antibody in zQ175/+ mice injected with Cas13d-NT (**a**) or Cas13d-CAG^EX^ (**b**). Red, EM48 immunostaining; blue, DAPI nuclear staining. Scale bar, 10 µm. **c**, Quantification of mHTT aggregates in the zQ175 mice injected with AAV9–Cas13d–NT or AAV9–Cas13d–CAG^EX^. *P < 0.05 by two-sided Student’s *t*-test. *n* = 4 mice per group, 2 females and 2 males per group, 10 months of age. *P* = 0.0261. **d**, Quantification of mutant and WT mRNA via allele-specific RT–qPCR in Q175/+ mice treated with either Cas13d–NT or Cas13d–CAG^EX^ (two-way ANOVA, Tukey post hoc test, ****P < 0.0001, *n* = 4 per experimental group). **e**,**f**, Western blot analysis of mutant HTT, WT HTT and HA (Cas13d constructs containing HA tag) protein levels in zQ175/+ (**e**) and WT (**f**) mice treated with either Cas13d–NT or Cas13d–CAG^EX^, *n* = 3 mice/group. **g**–**k**, Quantification of mutant HTT (**g**), WT HTT (**h**,**i**) and HA (**j**,**k**) protein levels in WT and zQ175/+ mice treated with either Cas13d–NT or Cas13d–CAG^EX^ (*n* = 3 biological replicates per experimental group). One-sided Student’s *t*-test. NS, not significant; *P < 0.05. *P* = 0.03 in Fig. 5g. No adjustment was made for multiple comparisons. Source data

We next determined the effect of Cas13d–CAG^EX^ on mHTT aggregation in zQ175/+ mouse striatum. The Cas13d–CAG^EX^-injected striatum of zQ175/+ mice harbored significantly reduced EM48^+^ mutant aggregates compared with those injected with Cas13d–NT (Fig. 5a–c), indicating that Cas13d–CAG^EX^ can effectively suppress mutant HTT aggregates in vivo. Importantly, we observed a significant reduction of mHTT *HTT* and protein levels in the striatum of Cas13d–CAG^EX^ -injected zQ175/+ mice. WT mouse HTT mRNA and protein levels, which could be distinguished from the mutant human exon-containing *HTT* allele, were preserved in both zQ175/+ and WT mice treated with Cas13d–CAG^EX^ (Fig. 5d–f quantified in Fig. 5g–k). This once again demonstrates the potential for allele-selective targeting of pathogenic HTT mRNA using Cas13d–CAG^EX^.

**Extended Data Fig. 5:**
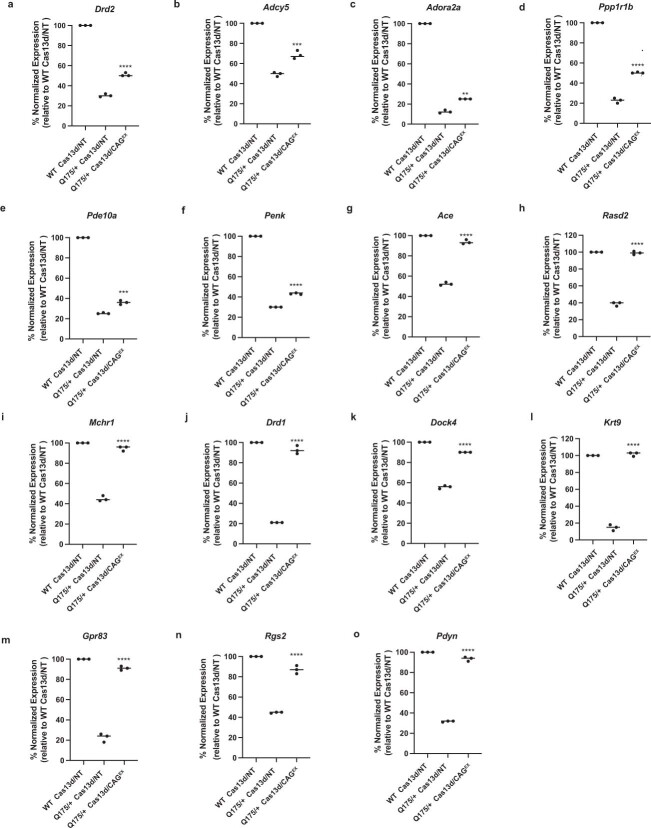
Cas13d/CAG^EX^ leads to partial reversal of striatum-specific HD disease markers in Q175/+ mice. **a-o**. Quantification of striatum-specific HD disease markers via quantitative PCR. CAG^EX^ (one-way ANOVA, Tukey Posthoc Test,, **p = 0.046, (b)*** p = 0.0003, (e)*** p = 0.0002, ****p < 0.0001; n = 3 per experimental group). Source data

We next performed transcriptome-wide analyses on striatal samples isolated from zQ175/+ and WT control mice treated with Cas13d–CAG^EX^ or Cas13d–NT. In doing so, we identified widespread transcriptome dysregulation caused by m*HTT* with a total of 2,413 downregulated and 2,368 upregulated DEGs in the Cas13d–NT-treated zQ175/+ mice compared to WT littermates (FDR-adjusted *P* *<* 0.01) (Supplementary Table 6). GO analysis showed that many of our DEGs in zQ175/+ mice are involved in ‘cAMP signaling’, ‘protein phosphorylation’ and ‘negative regulation of cell cycle transition’. These results resonate with previous reports^48,49^ and include a large panel of established striatum-specific markers of HD (Fisher’s exact test with FDR-adjusted *P* < 0.05; Extended Data Fig. 5a and Supplementary Table 7). Indeed, we observed statistically significant overlaps between our DEGs from our Q175/+ studies and those previously reported^48,49^ (hypergeometric *P* < 0.0001; Extended Data Fig. 5b and Supplementary Tables 8 and 9).

**Fig. 6:**
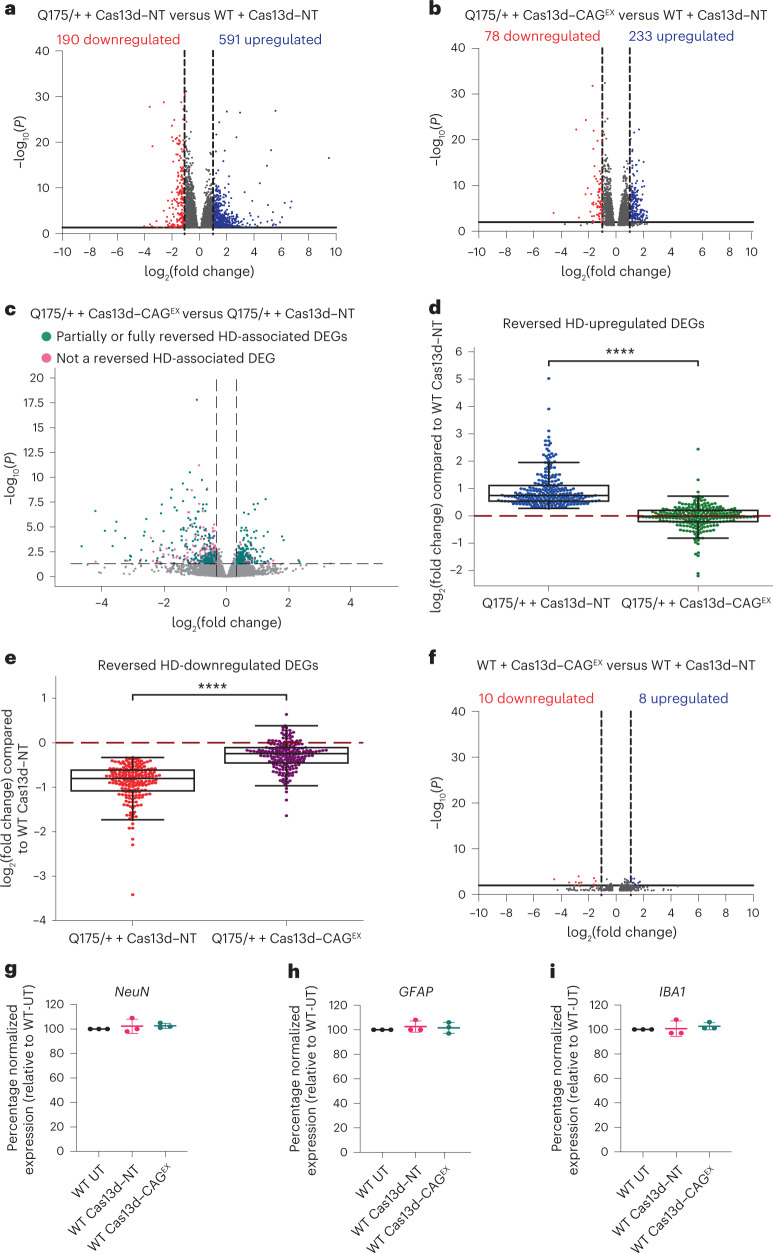
Cas13d–CAG^EX^ partially reverses HD-associated differential gene expression in a full-length knock-in mouse model of HD. Cas13d–CAG^EX^ or Cas13d–NT was injected into the striatum of two-month-old HD zQ175 or WT mice and the striatum was collected at ten months of age. Total RNA was extracted and quantified by RNA-seq. *n* = 4 or 5 mice per experimental group. **a**,**b**, Scatter plots of upregulated (**a**) and downregulated (**b**) DEGs in zQ175/+ mice treated with either Cas13d–NT or Cas13d–CAG^EX^ AAV9 showing partial reversal of HD-mediated transcriptome changes in HD mouse striatum by Cas13d–CAG^EX^. **c**, Volcano plot for DEGs between Q175 + Cas13d–CAG^EX^ versus Q175 + Cas13d–NT. Most DEGs correspond to a partial to full reversal of HD-associated DEGs (green). Fold change is relative to Q175 + Cas13d–NT. Significance cutoffs are fold change greater ±20% and FDR-adjusted *P* < 0.05. **d**,**e**, Averaged expression of HD-associated upregulated (**d**) or downregulated (**e**) DEGs that were significantly reversed by Cas13d–CAG^EX^ treatment. The red dashed line at 0 indicates full rescue to WT levels. ****P < 0.0001, two-sample paired Wilcoxon signed-ranks test (*n* = 4 mice per experimental group). The box extends from the first quartile to the third quartile of the data with a line at the median. The whiskers extend from the box by 1.5× the interquartile range. Flier points are those past the end of the whiskers. **f**, Scatter plot of upregulated and downregulated DEGs in WT mice treated with either Cas13d–NT or Cas13d–CAG^EX^ AAV9 showing limited off-targets. **g**–**i**, Quantification of CNS-specific markers of immunogenicity via qPCR (NeuN (**g**), GFAP (**h**), and IBA1 (**i**)) (one-way ANOVA, Tukey post hoc test, *n* = 3 per experimental group; *n* = 1). Data are presented as mean values ± s.e.m. Source data

**Extended Data Fig. 6:**
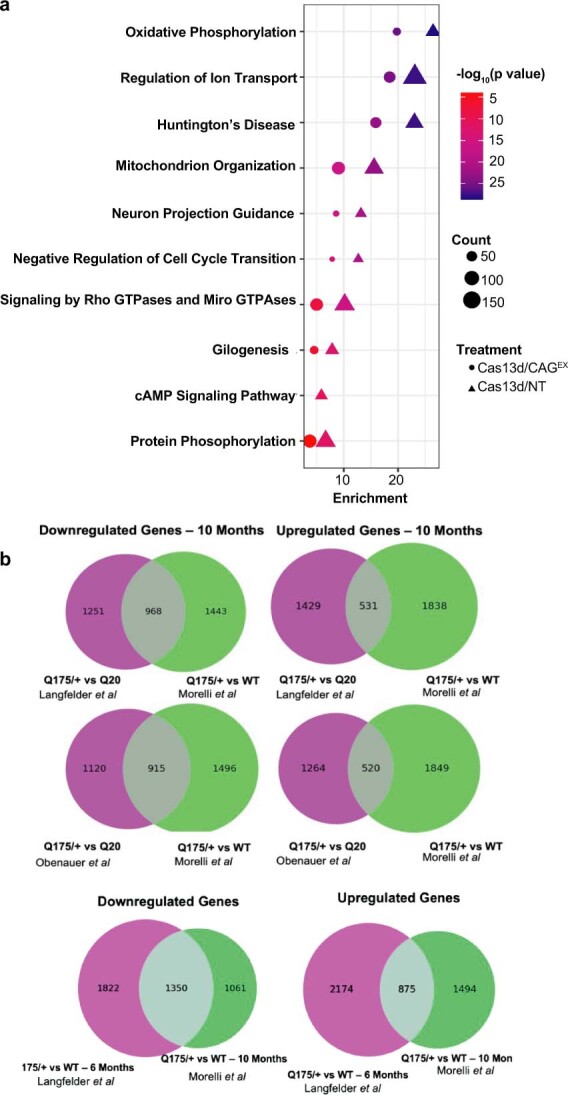
Cas13d/CAG^EX^ partially reverses HD-mediated biomarkers in Q175/+ mice. **a**. GO analysis of upregulated and downregulated DEGs in zQ175/+ as well as HD DEGs reversed by Cas13d/CAG^EX^. Significant GO terms were determined by Fisher’s exact test after FDR correction at p < 0.05 and sorted by fold enrichment. **b**. Overlay of reported Q175/+ DEGs in *Morelli et al* and those previously reported in *Landfelder et al* and Obenauer et al with a significance threshold of FDR-adjusted p-value < 0.01.

We next determined that approximately 56% of these m*HTT*-mediated transcriptional changes identified in Q175/+ mice treated with Cas13d–NT (2,677 out of 4,781 DEGs) were reversed by Cas13d–CAG^EX^ treatment with a fold change of 50% or more toward WT levels (satisfying an FDR-adjusted *P* threshold of <0.01; Supplementary Table 6). Excitingly, we also found that 60.2% of the most robust HD DEGs (twofold change, FDR-adjusted *P* < 0.001) were partially to fully reversed, including most of previously reported striatum-specific HD disease markers identified in our control-treated zQ175/+ mice (59 out of 74; 79.7%; Fig. 6a,b, Supplementary Table 10 and Extended Data Fig. 5a–o). The DEGs that were corrected were evenly enriched for each HD-associated pathological pathway identified in our zQ175/+ mice treated with Cas13d–NT, supporting that our approach can mitigate some known downstream effects of m*HTT* (Extended Data Fig. 6a and Supplementary Table 8). Furthermore, when comparing zQ175/+ mice treated with Cas13d–CAG^EX^ to ones treated with Cas13d–NT, 66.5% (189 out of 284) of upregulated DEGs and 54.0% (238/441) of downregulated DEGs partially to fully reversed to resemble WT levels, that is, if they exhibited a significant reduction in difference between their zQ175/+ and WT levels (FDR-adjusted *P* < 0.01, difference of more than 20% and depicted in Fig. 6c; Supplementary Table 11). Excitingly, some genes achieved almost complete rescue (*P* < 10^−4^, Wilcoxon two-sample paired signed-rank test; Fig. 6d,e).

**Extended Data Fig. 7:**
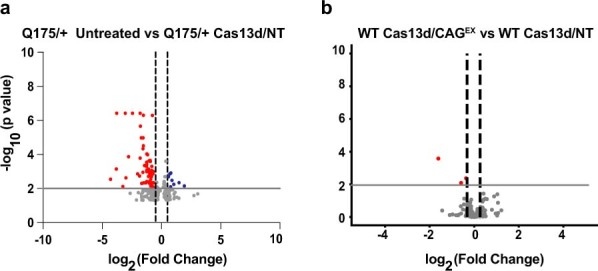
Cas13d/CAG^EX^ causes limited off-target effects on the mouse transcriptome *in vivo*. **a**. Scatter plot of CAG-expanded transcripts in the mouse transcriptome within Q175/+ mice treated with either Cas13d/NT or Cas13d/CAG^EX^ (FDR-adjusted p-value < 0.01). **b**. Scatter plot of CAG-expanded transcripts in the mouse transcriptome within WT mice treated with either Cas13d/NT or Cas13d/CAG^EX^ (FDR-adjusted p-value < 0.01).

We next evaluated the safety profile of Cas13d–CAG^EX^ based on our RNA-seq data. We observed that approximately 80 genes are differentially expressed when comparing Cas13d–NT-treated and untreated zQ175/+ mice, which may be a result of injection, the AAV9 viral delivery vector, Cas13d, the gRNA or a combination of these factors (Extended Data Fig. 7a). Nevertheless, when comparing WT mice treated with Cas13d–CAG^EX^ or Cas13d–NT, we found only 18 statistically significant (FDR-adjusted *P* < 0.01) changes in gene expression (Fig. 6f). We then examined the expression levels of protein-coding transcripts with five or more non-disease-associated CAG, AGC or GCA tandem repeats, which could be inadvertently targeted by the CAG^EX^ gRNA. Of 344 transcripts with 5 or more repeats, only 3 significant DEGs (FDR-adjusted *P* < 0.01) were detected (Extended Data Fig. 7b and Supplementary Table 12). Furthermore, qPCR also confirmed that Cas13d–CAG^EX^ did not disrupt CNS-specific markers of immunogenicity including neuronal nuclei (NeuN), glial fibrillary acidic protein (GFAP) and ionized calcium binding adaptor molecule 1 (IBA1) (Fig. 6g–i). Taken together, these results support the efficacy and safety of Cas13d–CAG^EX^ in HD preclinical models.

## Discussion

We packaged *R. flavefaciens XPD3002* Cas13d, an RNA-targeting enzyme 2.4 kilobases of DNA in size, and a CAG^EX^ RNA-targeting gRNA in a single viral delivery vehicle. We determined that Cas13d–CAG^EX^ selectively reduces CAG^EX^ *HTT* RNA in striatal neurons derived from the iPSC lines of three patients with HD with CAG expansions ranging from 66 to 109 STRs and patient fibroblasts with 46 and 67 CAG repeats in *HTT*, suggesting potential therapeutic benefit for both adult and juvenile patient populations with HD. Viral transduction of Cas13d–CAG^EX^ in premanifest zQ175/+ HD mice resulted in allele-selective suppression of mutant HTT mRNA and protein with minimal off-targets. Lastly, we demonstrated significantly improved motor function, reduced m*HTT* aggregates and attenuated striatal atrophy in Cas13d–CAG^EX^-treated zQ175/+ HD mice.

Approximately, 2.7 out of 100,000 individuals worldwide currently manifest symptoms of HD^50^. Unfortunately, to date, neither curative nor disease-modifying treatments for HD exist. Current therapeutic strategies target different aspects of HD pathology including excitotoxicity, the dopamine pathway, caspases, mHTT protein aggregation, mitochondrial dysfunction and transcriptional dysregulation^51^. Unfortunately, the only therapeutics currently approved by the U. S. Food and Drug Administration (FDA) only alleviate symptoms and do not modify disease progression. Because the molecular etiology of HD is complex, one accepted therapeutic approach for all patient populations suffering from HD includes the selective elimination of mutant HTT (RNA and protein) produced by this locus^52^. Current strategies to attenuate gene expression via targeted destruction of mHTT RNA include ASOs, RNAi compounds and small molecule splicing modulators. The most promising of these include the Roche phase III ASO candidate tominersen and the Wave Therapeutics phase I/II ASO candidates WVE-120101 and WVE-120102 (ref. ^53^). However, each failed to show adequate efficacy and safety in their respective clinical trials. Preliminary results from clinical trial NCT03761849 indicated that two independent dosing regimens of tominersen in patients performed slightly worse than placebo on the primary outcome measures of the Composite Unified Huntington Disease Rating Scale and Total Functional Capacity^26,54^. Revised clinical trials will be conducted to determine if these outcomes resulted from either the repeated high dose used or interference with WT HTT protein levels^26,54^. Wave’s ASO candidates that target patient-specific single-nucleotide polymorphisms on mHTT pre-mRNA showed limited target specificity, which led to low efficacy.

To identify an optimal gRNA against CAG expansions, we profiled three different Cas13d gRNAs that target the three possible sequence frames in the CAG repeat tract. One directly in-frame to CAG expansions (CTG-) and two with one or two bases out of frame were constructed and screened for CAG^EX^ RNA knockdown efficiency with our TGC-targeting gRNA surprisingly yielding the most effective depletion of the CAG. This was a critical optimization step since knockdown efficiency of RNA transcripts by each individual RNA-targeting CRISPR system is highly dependent on gRNA-specific features and target site context. Since *Rfx*–Cas13d is still a newly characterized RNA-targeting system, the molecular engineering field is in the process of understanding the targeting parameters and sequence constraints for optimal gRNA design, which is especially important for double-stranded RNA structures since hairpins form from CAG expansions. Indeed, mRNA stability largely depends on the mRNA nucleotide sequence, which affects the secondary and tertiary structures of mRNAs and the accessibility of various RNA-binding proteins to the mRNA^55^. Abnormal expansions of CAG repetitive elements within RNA form stable secondary structures that consists of a base, a hairpin structure forming the stem and a terminal loop with the overall thermodynamic stability of CAG-expanded mRNA transcripts increasing with CAG repeat length^56–60^. Such stable GC-rich RNA hairpin structures have been widely known to sequester RNA-binding proteins that have an increased binding affinity for hairpin loops formed from expanded STRs compared to their canonical targets^8,61^, suggesting that Cas13d may also have a similar binding preference. Interestingly, the observed specificity of our Cas13d system for long, pathogenic repeats that form RNA hairpins has also been observed in recent studies that involve RNA-targeting CRISPR–Cas9 systems programmed to degrade microsatellite repeat expansions in vitro^30^ and in vivo^31^ with a similar gRNA design.

Excitingly, we demonstrate that administration of Cas13d–CAG^EX^ in premanifest zQ175/+ HD mice resulted in allele-selective suppression of mutant HTT mRNA and protein while maintaining normal mRNA and protein levels with limited off-target effects on the mouse transcriptome. This is an improvement over current RNA-targeting approaches, including small molecules with limited target specificity compared to Watson–Crick base pairing and RNAi compounds that may deplete the endogenous RNAi machinery and cause undesired global alterations in gene expression. We also showed safe and long-lasting therapeutic efficacy eight months postinjection from AAV9-mediated delivery of Cas13d–CAG^EX^ to the striatum of zQ175/+ HD mice. AAV9 is an FDA-approved gene therapy delivery method that affords sustained transgene expression and is currently being used in several clinical trials. On the other hand, ASOs and other small molecules must be continually readministered for the lifetime of the patient, which can pose safety issues in the affected CNS tissues. However, such transient therapeutic effects of ASOs and small molecules can be seen as an advantage over AAV drug delivery because their administration can be halted if negative side effects are observed at any point during a patient’s lifetime and such adverse effects are likely to be reversible.

Taken together, we demonstrate a proof of principle for a CAG^EX^ RNA-targeting CRISPR–Cas13d system as a potential allele-sensitive therapeutic approach for HD, a strategy with broad implications for the treatment of other neurodegenerative disorders.

## Methods

### Animals

Heterozygous zQ175 (zQ175/+) mice and WT littermates (both sex- and age-matched in each group) were used in the study. The zQ175 line in C57BL/6 background strain was obtained from The Jackson Laboratory and bred and maintained in our laboratory. Genotyping and CAG repeat size were determined at Laragen by PCR of tail snips. The CAG repeat length was 223 ± 3 in male zQ175/+ mice and 225 ± 3 in female zQ175 mice/+ used in the study. All mice were housed under specific pathogen-free conditions with a reversed 12-h light–dark cycle maintained at 23 ˚C with 30–70% humidity and provided with food and water ad libitum. All behavioral tests and longitudinal MRI measures were done in the mouse dark phase (active). The study was carried out in strict accordance with the recommendations in the Guide for the Care and Use of Laboratory Animals of the National Institutes of Health and approved by the Animal Care and Use Committee at Johns Hopkins University. A rigorous study design was implemented. Mice were randomized into groups to avoid bias. A balanced sex ratio was considered in all experiments. Data were collected using animal IDs and analyzed by investigators who were blinded to genotype and treatment. Data from different sexes were analyzed grouped when there was no sex-dependent difference in the outcome measures.

### Cell lines

Human iPSC lines derived from individuals with HD and neurotypical controls have been previously characterized elsewhere^32–34^. HD stem cell lines 66, 77 and 109 were a gift from the L. Thompson’s laboratory at UC Irvine whereas our neurotypical controls were a gift from the Goldstein laboratory at UCSD. iPSC colonies were expanded on Matrigel-coated dishes (BD Biosciences) with mTeSR1 medium (STEMCELL Technologies). Cells were routinely checked by karyotype and CNV arrays to avoid genomic alterations in the culture. They were purchased from the American Type Culture Collection and were not further authenticated. HEK293T cells were obtained from Millipore Sigma (catalog no. 12022001). HD human fibroblast lines GM04723 (CAG15/67) and GM02151 (CAG18/46) were obtained from the Coriell Cell Repository.

### Cas13d–CAG^EX^ design strategy

We designed Rfx CRISPR–Cas13d gRNAs adapted from Konermann et al.^28^ to be complementary to CAG^EX^ and packaged the RNA-targeting Cas13d system into a single AAV9 vector or lentiviral vector termed ‘Cas13d–CAG^EX^’. Three gRNAs, one directly in-frame to CAG expansions (CTGCTG….) and the other two 1 or 2 bases out of frame where (TGC…. and GCT….) were constructed and screened for expanded-CAG RNA knockdown efficiency.

The sequence of gRNAs was: CAG^EX^-1 gRNA GTCGTCGTCGTCGTCGTCGTC; CAG^EX^-2 gRNA TCGTCGTCGTCGTCGTCGTCG; and CAG^EX^-3 gRNA CGTCGTCGTCGTCGTCGTCGT.

### Transfections

HEK293 cells were plated at a density of 3 × 10^5^ cells per well of a 6-well plate for RNA isolation. For RNA isolation, 150 ng of a CAG^105^ plasmid that expressed 105 CAG repeats was used with 500 ng each of plasmids containing both Cas13d and 1 of the 3 CAG-targeting guides. Transfection was carried out using Lipofectamine 3000 according to the manufacturer’s instructions. Cells were incubated for 72 h at 37 °C and 5% CO_2_.

### PolyQ western blot

Protein lysates were resolved on 4–15% Mini-PROTEAN Tris-Glycine gels (Bio-Rad) and transferred to an Invitrolon and Immobilon-P polyvinylidene fluoride membrane. Membranes were blocked with 5% skimmed milk in TBST (1× Tris-buffered saline, 0.1% Tween 20) and incubated overnight with primary antibodies: anti-polyQ disease protein antibody, 1:1,000, Merck Millipore MAB1574 (clone 5TF1-1C2) and anti-beta tubulin, 1:5,000, catalog no. ab6046 (Abcam) diluted in blocking solution at 4 °C. After three 10-min washes in TBST, the blots were incubated with the appropriate horseradish peroxidase (HRP)-conjugated secondary antibodies (anti-rabbit IgG (catalog no. NEF812001) and anti-mouse IgG (catalog no. NEF822001EA); PerkinElmer) diluted in blocking solution. After three 10-min washes in TBST, the blots were developed using Western Lightning Plus-ECL enhanced chemiluminescence substrate (PerkinElmer). Phosphorylation of protein was detected via the addition of alkaline phosphatase to the protein lysate (1:1,000, catalog no. 10567752001; Merck Millipore) before to SDS–polyacrylamide gel electrophoresis.

### Allele-specific RT–qPCR in the fibroblasts of patients with HD

The Huntington’s disease fibroblast lines GM04723 (CAG15/67) and GM02151 (CAG18/46) were obtained from the Coriell Cell Repository and maintained in complete minimal essential medium with 20% FCS. CAG repeat lengths were confirmed by sequencing.

mRNA transfection was performed using a 4D-Nucleofector X Kit (Lonza). We transfected 1 µg Cas13d–NT or Cas13d–CAG^EX^ per 5 × 10^5^ cells. Seventy-two hours after transfection, cells were collected for gene expression analysis by RT–qPCR. RNA was purified using RNeasy columns (QIAGEN) and converted to complementary DNA (cDNA) using the High-Capacity cDNA Reverse Transcription Kit (Thermo Fisher Scientific). Allele-specific detection of human *HTT* expression in HD fibroblasts GM04723 (CAG15/67) and GM02151 (CAG18/46) was performed with the PrimeTime Gene Expression Master Mix (Integrated DNA Technologies) as well as Integrated DNA Technologies PrimeTime qPCR assays adapted from Zeitler et al.^62^ with a probe to a primer ratio of 2:1 based on SNP rs363099-C/T (exon 29): 363099C-forward: AGTTTGGAGGGTTTCTC; 363099T-forward: AGTTTGGAGGGTTTCTT; 363099T-blocker: AGGGTTTCTCCGCTCAGC/phos/; 363099-common reverse: TCGACTAAAGCAGGATTTCAGG.

### Differentiation of iPSCs into two-dimensional neuronal cultures containing enrichment for striatal neurons

Differentiations were performed as follows: iPSC colonies were washed with PBS, pH 7.4 (Gibco) and then switched to neural induction medium (advanced DMEM/F12 (1:1) supplemented with 2 mM GlutaMAX (Gibco), 2% B27 without vitamin A (Thermo Fisher Scientific), 10 μM SB431542 and 1 μM LDN 193189 (both STEMCELL Technologies) and 1.5 μM IWR1 (Tocris)) with daily medium changes; this was day 0. On day 4 cells were passaged 1:2 with Stempro Accutase (Invitrogen) for 5 min at 37 °C and replated on human embryonic stem cell (hESC)-qualified Matrigel. At day 8, cells were passaged again 1:2 with Stempro Accutase for 5 min at 37 °C and replated on hESC-qualified Matrigel in a different medium (advanced DMEM/F12 (1:1) supplemented with 2 mM GlutaMAX, 2% B27 without vitamin A, 0.2 μM LDN 193189, 1.5 μM IWR1, 20 ng ml^−1^ Activin A; PeproTech). At day 16, cells were plated for neuronal differentiation in SCM1 medium (advanced DMEM/F12 (1:1) supplemented with 2 mM GlutaMAX, 2% B27 (Invitrogen), 10 μM DAPT, 10 μM Forskolin, 300 μM γ-aminobutyric acid, 3 μM CHIR99021, 2 μM PD 0332991 (all Tocris), to 1.8 mM CaCl_2_, 200 μM ascorbic acid (Sigma-Aldrich), 10 ng ml^−1^ brain-derived neurotrophic factor (BDNF) (PeproTech), medium was 50% changed every 2–3 d. On day 23, there was a full medium change to SCM2 medium (advanced DMEM/F12 (1:1): neurobasal A (Gibco) (50:50) supplemented with 2 mM GlutaMAX), 2% B27, to 1.8 mM CaCl_2_, 3 μM CHIR99021, 2 μM PD 0332991, 200 μM ascorbic acid, 10 ng ml^−1^ BDNF) and medium was 50% changed every 2–3 d until day 32 for collection^32–34^.

### *Mycoplasma* testing

All iPSC and striatal neuron cultures were routinely tested for *Mycoplasma* by PCR. Media supernatants (with no antibiotics) were collected, centrifuged and resuspended in saline buffer. Ten microliters of each sample were used for PCR with the following primers: forward: GGCGAATGGGTGAGTAAC; reverse: CGGATAACGCTTGCGACCT. Only negative samples were used in the study.

### Lentivirus production

Both Cas13–CAG^EX^ and Cas13d–NT constructs were cotransfected with psPAX2 (packaging plasmid) (Addgene plasmid no. 12260) and VSV-G (viral envelope) into Lenti-X HEK293T cells (Clontech) at a mass ratio of 5:4:3 using polyethyleneimine. Viral-containing supernatants were collected and concentrated with Lenti-X Concentrator reagent (Clontech) according to the manufacturer’s protocol (100× concentration) and aliquoted for storage.

### Immunocytochemistry

iPSC-derived neurons were permeabilized with 0.3% Triton X (Sigma-Aldrich) in PBS for 10 min and then blocked for 30 min in 5% goat serum 2.5% BSA (Gibco) 0.1% Triton X in PBS. Samples were incubated with primary antibodies overnight at 4 °C (DARPP-32, 1:500, catalog no. ab40801 (EP720Y) (Abcam); CTIP2, 1:500, catalog no. ab18465 (25B6) (Abcam); anti-huntingtin protein antibody 1:200, catalog no. MAB5347 (mEM48) (Merck Millipore)) and then washed 3 times with PBS before an overnight 4 °C incubation in secondary antibody (Alexa Fluor rabbit 555 IgG (catalog no. A-31572; Invitrogen), rat 488 IgG (catalog no. A48262; Invitrogen) and mouse 488 IgG (catalog no. A32723; Invitrogen) for 1 h at 1:500 and subsequently incubated with Hoechst 33342 (Sigma-Aldrich) and mounted with VECTASHIELD. Samples were imaged with a Zeiss LSM 780 confocal microscope at 20×.

### Human moesin bulk RNA-seq library generation

RNA was isolated from approximately 100,000 moesin (MSN) cells from 4 independent differentiations per experimental group according to the manufacturer’s protocol and purified using RNeasy columns. A total of 72 mRNA libraries for Illumina sequencing were prepared using the KAPA mRNA HyperPrep Kit (catalog no. KK8581; KAPA Biosystems) from 250 ng of total RNA. Libraries were sequenced on an Illumina NovaSeq instrument to a minimum of 3 × 10^7^ paired-end 100-base pair (bp) reads.

### Human MSN RNA-seq data processing and analysis

RNA-seq reads were adapter-trimmed using Cutadapt v.1.14 and mapped to human-specific repetitive elements from RepBase v.18.05 by STAR v.2.4.0i^63^. Repeat-mapping reads were removed and remaining reads were mapped to the human genome assembly (hg38) with STAR. Read counts for all genes annotated in GENCODE v.24 (hg38) were calculated using the read summarization program featureCounts (v. 2.0.2)^64^ (coding sequence regions only). Differential expression analysis between different experimental groups (HD untreated, HD Cas13d–NT, HD Cas13d–CAG^EX^, control 1–3 untreated, control 1–3 Cas13d–NT, control 1–3 Cas13d–CAG^EX^) was performed using DESeq2 (v.1.38.1)^35^. Differentially expressed mRNAs were defined as having an FDR-adjusted *P* < 0.01 as determined by the Wald test, the standard statistical test used by DESeq2^35^. We used the Unix grep search tool to scan the human genome for all protein-coding transcripts with five or more consecutive CAG, AGC and GCA tandem repeats.

### RNA dot blot

HEK293 cell and human MSN RNA quality and concentrations were estimated using the NanoDrop spectrophotometer. Then, 5 μg of total RNA was resuspended in 1 mM EDTA, pH 8.0 to a final volume of 50 μl followed by the addition of 30 μl 20× SSC and 20 μl 37% formaldehyde to denature the RNA. The RNA was incubated for 30 min at 60 °C followed by a 2-min incubation on ice. The Bio-Rad Bio-Dot apparatus was assembled according to the manufacturer’s protocol with Hybond N+ nylon membrane (GE Healthcare) on the top. The membrane was equilibrated by passing 100 μl of 20× SSC through the slots using a vacuum. The denatured RNA was passed through the slots and the membrane was washed with 20× SSC. The membrane was crosslinked at the auto setting in the UV Stratalinker (Stratagene). ULTRAhyb ultrasensitive hybridization buffer prewarmed at 37 °C was used to pretreat the membrane for 10 min at 37 °C. A custom biotinylated probe (CTGCTGCTGCTGCTGCTGCTGCTG-3′biotin (Integrated DNA Technologies)) was added to the prehybridization solution (5 μl ml^−1^). Hybridization was conducted for 5 h at 37 °C and the membrane was washed once with 1× SSC for 10 min at 37 °C. The membrane was exposed using the Chemiluminescent Nucleic Acid Detection Module Kit (Thermo Fisher Scientific).

### AAV packaging

Cas13d–CAG^EX^ (no. 1457 Cas13d–CAG^EX^: 3.9 × 10^12^ vg ml^−1^) and Cas13d–NT (no. 1457 Cas13d–NT: 4.4 × 10^12^ vg ml^−1^) were cloned into an AAV vector provided by the UCSD Viral Vector Core. Each vector was packaged into single-stranded AAV9 by both the UCSD Viral Vector Core and the Emory Viral Vector Core (Emory University) using standard procedures and quality controls established at each independent core.

### Stereotaxic injection

The mice were anesthetized with 1.5% isoflurane inhalation and stabilized in a stereotaxic instrument (David Kopf Instruments). Mice were injected into the striatum using the following stereotaxic coordinates: 0.62 mm rostral to bregma; ±1.75 mm lateral to midline; and 3.5 mm ventral to the skull surface. Then, 1 μl of a Cas13d–CAG^EX^-containing AAV9 titer (3.9 × 10^12^ vg ml^−1^) or Cas13d–NT-containing AAV9 titer (4.4 × 10^12^ vg ml^−1^, diluted to 3.9 × 10^12^ vg ml^−1^) were injected into the striatum using a Hamilton syringe infusion pump (World Precision Instruments) at a perfusion speed of 200 nl min^−1^.

### Immunohistochemistry and quantification

Mice were anesthetized and perfused transcardially with PBS followed by 4% paraformaldehyde. Brains were postfixed overnight followed by immersion in 30% sucrose for 24 h. Frozen sections (thickness, 10–15 μm) of coronal brain were immunostained with the following antibody: EM48 (1:200, catalog no. MAB5347; Merck Millipore). Briefly, the sections were washed three times with PBS, then permeabilized by incubating with 0.3% Triton X-100 for 5 min, followed by incubation with blocking solution containing 5% donkey serum or 3% goat serum and 0.3% Triton X-100 for 1 h. The sections were then incubated with primary antibody at 4 °C overnight. After three washes with PBS, the sections were incubated with fluorescence-labeled secondary antibody (Alexa Fluor mouse 555 IgG (catalog no. A32727, Invitrogen)) for 2 h at room temperature. Sections were mounted onto Superfrost slides (Thermo Fisher Scientific) dried and then covered with antifade mounting solution. Fluorescence images were acquired with a Keyence BZ-X700 All-in-One florescence microscope.

For image analysis, the samples were coded with ID and images were analyzed by investigators who were blinded to the genotypes and treatment. The results of the analysis were then calculated statistically and decoded by different investigators at the end. The results from three microscopy fields per slide and three sections per mouse were calculated for each mouse brain. EM48-positive puncta quantities were determined using the ImageJ (version 1.53t) (Fiji) analyze particles plugin function. The numbers of EM48-positive puncta per mm^2^ were determined at a constant threshold for each stain using ×20 images for quantifications.

### Mouse *mHtt* RT–qPCR

For qPCR, RNA was converted to cDNA using the High-Capacity cDNA Reverse Transcription Kit (Thermo Fisher Scientific). Quantification of mRNA in mouse tissue samples was completed with the PrimeTime Gene Expression Master Mix (Integrated DNA Technologies) and Integrated DNA Technologies PrimeTime qPCR assays adapted from Kingwell^54^.

The forward primer for the WT *Htt* allele was CAGGTCCGGCAGAGGAACC. The forward primer for the Q175 *mHtt* allele was GCCCGGCTGTGGCTGA. The reverse primer for both the WT and Q175 *Htt* alleles was TTCACACGGTCTTTCTTGGTGG. The probe for both the WT and Q175/+ *Htt* alleles was TGCACCGACCAAAGAAGGAACTCT.

Total mouse *Htt* was quantified with the Integrated DNA Technologies assay Mm.PT.58.12088552 while the following Integrated DNA Technologies PrimeTime qPCR assay were used to quantify striatal-specific markers of HD in Extended Data Fig. 6: *Drd2*: Mm.PT.58.7811767; *Adcy5*: Mm.PT.58.37585706; *Adora2a*: Mm.PT.58.29505675; *Ppp1r1b*: Mm.PT.58.8281675; *Pde10a*: Mm.PT.58.17658388; *Penk*: Mm.PT.58.45924319; *Ace*: Mm.PT.58.43658045; *Rasd2*: Mm.PT.58.33380601; *Mchr1*: Mm.PT.58.10781964; *Drd1*: Mm.PT.58.43576955.g; *Dock4*: Mm.PT.58.11402098; *rt9*: Mm.PT.58.8514559; *Gpr83*: Mm.PT.58.9029332; *Rgs2*: Mm.PT.58.41856362; *Pdyn*: Mm.PT.58.29316720.

Mouse *Actn2* Integrated DNA Technologies PrimeTime qPCR assay Mm.PT.58.28953379 was used as an internal housekeeping control.

### Western blotting

Striatal samples were homogenized in a buffer containing 50 mM Tris-HCl (pH 8.0), 150 mM NaCl, 0.1% (w/v) SDS, 1.0% NP-40, 0.5% sodium deoxycholate and 1% (v/v) protease inhibitors. Then, 30 μg of proteins were separated in a 4–20% gradient gel and transferred to a nitrocellulose membrane. The membrane was blotted with the following primary antibodies: anti-HTT, 1:1,000, catalog no. MA2166 (clone 1HU-4C8) (Merck Millipore); anti-polyQ, 1:1,000, catalog no. MABN2427 (clone MW1) (Merck Millipore); and β-actin, 1:5,000, catalog no. A5316 (clone AC-74) (Sigma-Aldrich). After incubation with HRP-conjugated secondary antibodies (anti-rabbit IgG NEF812001 and anti-mouse IgG NEF822001EA) bands were visualized by chemiluminescence.

### In vivo structural MRI acquisition

In vivo MRI was performed on a vertical 9.4 Tesla MR scanner (Bruker Biospin) with a triple-axis gradient and a physiological monitoring system (electrocardiogram, respiration and body temperature). Mice were anesthetized with isoflurane (1%) mixed with oxygen and air at 1:3 ratios via a vaporizer and a facial mask and scanned longitudinally (the same mice were imaged repeatedly over a 12-month period). We used a 20-mm diameter volume coil as the radiofrequency transmitter and receiver. Temperature was maintained by a heating block built into the gradient system. Respiration was monitored throughout the entire scan.

High-resolution anatomical images were acquired by using a three-dimensional (3D) T2-weighted fast spin echo sequence with the following parameters: echo time (TE)/repetition time (TR) = 40/700 ms; resolution = 0.1 × 0.1 × 0.25 mm; echo train length = 4; number of average = 2; and flip angle = 40°. Multislice T2-weighted images of the mouse brain were acquired by the rapid acquisition with refocused echoes (RARE) sequence with the following parameter—echo time (TE)/repetition time (TR) = 40 ms/1,500 ms, RARE factor = 8, in-plane resolution = 0.125 × 0.125 mm, slice thickness = 1 mm, total imaging time <2 min)—and used for high-resolution anatomical imaging. Total imaging time was about 50 min per mouse. Mice recovered quickly once the anesthesia was turned off and all mice survived the imaging sessions.

### Structural MRI image analysis

Images were first rigidly aligned to a template image from an adult control mouse which was selected from one of the images acquired from age-matched littermate control mice (mouse had the medium brain volume among the control group), which had been manually adjusted to the orientation defined by the Paxinos atlas with an isotropic resolution of 0.1 × 0.1 × 0.1 mm per pixel. After rigid alignment, images had the same position and orientation as the template image and image resolution was also adjusted to an isotropic resolution of 0.1 × 0.1 × 0.1 mm per pixel. Signals from non-brain tissue were removed manually (skull stripping). Skull-stripped, rigidly aligned images were analyzed using DiffeoMap (https://www.mristudio.org/). The registration accuracy was accessed by independent sets of labmarks that were manually placed at the brain contours. Intensity values of the gray matter, white matter and cerebrospinal fluid were normalized to the values in the template images by using a piece-wise linear function. This procedure ensured that subject and template images have similar intensity histograms. The intensity-normalized images were submitted by Landmarker software to a Linux cluster, which runs large deformation diffeomorphic metric mapping (LDDMM). The transformations were then used for quantitative measurement of changes in local tissue volume among different mouse brains by computing the Jacobian values of the transformations generated by LDDMM. There are 29 different brain regions segmented automatically.

### Behavioral tests

The 5-mm balance beam testing was conducted on an 80-cm-long and 5-mm-wide square-shaped balance beam that was mounted on supports 50-cm-high. A bright light illuminated the start platform and a darkened enclosed 1,728 cm^3^ escape box (12 × 12 × 12 cm^3^) was situated at the end of the beam. Mice were trained to walk across the beam twice at least 1 h before testing. The time for each mouse to traverse the balance beam was recorded with a 125-s maximum cutoff and falls were scored as 125 s.

### Mouse RNA isolation and RNA-seq library preparation

RNA was isolated from snap-frozen striatal mouse tissue from two independent cohorts of mice according to the manufacturer’s protocol and purified using RNeasy columns. A total of 13 mRNA libraries for Illumina sequencing were prepared using the KAPA mRNA HyperPrep Kit from 250 ng total RNA. Libraries were sequenced on an Illumina NovaSeq instrument to a minimum of 2 × 10^7^ paired-end 100-bp reads.

### Mouse RNA-seq data processing and analysis

To correct for batch effects between library preparations, ComBat-seq was applied to the raw read counts^65^. RNA-seq reads were adapter-trimmed using Cutadapt v.1.14 and mapped to mouse-specific repetitive elements from RepBase v.18.05 by STAR v.2.4.0i^64^. Repeat-mapping reads were removed and remaining reads were mapped to the mouse genome assembly (mm10) with STAR. Read counts for all protein-coding genes annotated in GENCODE v.M20 were calculated using the read summarization program featureCounts v.1.38.1^66^. Differential expression analysis was performed using DESeq2 v. 2.0.2^65^. Differentially expressed mRNAs were defined as having an FDR-adjusted *P* < 0.01 as determined by the Wald Test in the DESeq2 program. We used the Unix grep search tool to scan the mouse genome for all protein-coding transcripts with five or more consecutive CAG repeats.

### Human and mouse GO analysis

PANTHER v.16 was used for GO analysis^67^. All human or mouse protein-coding genes with at least five reads in any sample were used as the background set. Significant GO terms were determined by Fisher’s exact test after FDR correction at *P* < 0.05 and sorted by fold enrichment.

### Statistical analysis

Data are expressed as the mean ± s.e.m. unless otherwise noted. A Student’s *t*-test was used to measure the significant levels between WT or control and zQ175/+ or human HD groups at each given time point. Two-way repeated ANOVA (genotype and treatment) was performed to examine the statistical difference between different groups along with age and treatment. Statistical analysis was performed with Prism (GraphPad Software version 8.0.2). *P* values less than 0.05 were considered statistically significant (*n* is reported in the figure legends). Based on the means and s.d. from a previous study with Q175/+ mice^66^, a power analysis revealed that <3 mice reliably distinguish mutant and control populations (*P* < 0.05). To have 95% confidence in detecting an improvement with inhibition of the mutant *HTT* allele, 4 animals per genotype and treatment group are required (power analysis: two-way ANOVA, genotype × treatment, *P* ≤ 0.05). No statistical methods were used to predetermine sample sizes for the in vitro experiments but our sample sizes are similar to those reported in previous publications^30,33^. Data distribution was assumed to be normal but this was not formally tested for all experiments. Investigators were blinded to genotype and treatment both in vitro and in vivo experiments. No animals nor data points were excluded from the analyses for any reason. Neuronal cultures with <70% Cas13d transduction were eliminated from the study before outcome measures were taken. Mice were randomized into groups to avoid bias. Balanced sex ratio was considered in all experiments. Randomization was not available for the in vitro studies due to limited availability of patient lines and established neurotypical controls. All replications were successful and included in our outcome measures. Technical replication is represented by *n* in the figure legends.

### Reporting summary

Further information on research design is available in the Nature Portfolio Reporting Summary linked to this article.

## Online content

Any methods, additional references, Nature Portfolio reporting summaries, source data, extended data, supplementary information, acknowledgements, peer review information; details of author contributions and competing interests; and statements of data and code availability are available at 10.1038/s41593-022-01207-1.

## Supplementary information

Reporting Summary

Supplementary Table 1 List of common HD differentially expressed genes with patient iPSC-derived neuronal lines identified by transcriptome-wide RNA-seq.

Supplementary Table 2 GO analysis of human HD biomarkers.

Supplementary Table 3 List of patient-specific HD differentially expressed genes with HD patient iPSC-derived neuronal lines identified by transcriptome-wide RNA-seq.

Supplementary Table 4 Expression of human transcripts with five or more tandem CAG repeats in Cas13d/CAG^EX^-treated neuronal lines.

Supplementary Table 5 Striatum volumes (mm^3^).

Supplementary Table 6 List of HD differentially expressed genes in Q175/+ mice.

Supplementary Table 7 GO of Q175/+ HD biomarkers.

Supplementary Table 8 Overlap of Q175/+ differentially expressed genes within Morelli et al., Langfelder et al. and Obenauer et al.

Supplementary Table 9 Hypergeometric analysis of Q175/+ differentially expressed genes within Morelli et al., Langfelder et al. and Obenauer et al.

Supplementary Table 10 DE of striatum-specific HD disease markers in Q175/+ treated with Cas13d/CAG^EX^.

Supplementary Table 11 Partial to full reversal of HD biomarkers by Cas13d/CAG^EX^ in vivo.

Supplementary Table 12 Expression of mouse transcripts with five or more tandem CAG repeats in Cas13d/CAG^EX^-treated WT mice.

## Data Availability

All RNA-seq data discussed in this publication have been deposited in the Gene Expression Omnibus under accession no. GSE214110. There are no restrictions on data availability. Source data are provided with this paper.

## Source data

Source Data Fig. 1 Statistical source data.
Source Data Fig. 1 Unprocessed western blots.
Source Data Fig. 2 Statistical source data.
Source Data Fig. 4 Statistical source data.
Source Data Fig. 5 Statistical source data.
Source Data Fig. 5 Unprocessed western blots.
Source Data Fig. 6 Statistical source data.
Source Data Extended Data Fig. 1 Statistical source data.
Source Data Extended Data Fig. 2 Statistical source data.
Source Data Extended Data Fig. 3 Statistical source data.
Source Data Extended Data Fig. 4 Statistical source data.
Source Data Extended Data Fig. 5 Statistical source data.
