# Supplementary material for: An RNA-targeting CRISPR–Cas13d system alleviates disease-related phenotypes in Huntington’s disease models
Source: Nat Neurosci. 2022 Dec 12;26(1):27–38. doi: 10.1038/s41593-022-01207-1 (PMC9829537; doi:10.1038/s41593-022-01207-1)
Supplement: Supplementary file 1 — Reporting Summary [file 41593_2022_1207_MOESM1_ESM.pdf]

nature portfolio

Corresponding author(s): Drs. Gene Yeo and Wenzhen Duan

Last updated by author(s): 10/10/2022

Reporting Summary

Nature Portfolio wishes to improve the reproducibility of the work that we publish. This form provides structure for consistency and transparency in reporting. For further information on Nature Portfolio policies, see our Editorial Policies and the Editorial Policy Checklist.

Statistics

For all statistical analyses, confirm that the following items are present in the figure legend, table legend, main text, or Methods section.

n/a	Confirmed	
☐	☒	The exact sample size (*n*) for each experimental group/condition, given as a discrete number and unit of measurement
☐	☒	A statement on whether measurements were taken from distinct samples or whether the same sample was measured repeatedly
☐	☒	The statistical test(s) used AND whether they are one- or two-sided<br>*Only common tests should be described solely by name; describe more complex techniques in the Methods section.*
☐	☒	A description of all covariates tested
☒	☐	A description of any assumptions or corrections, such as tests of normality and adjustment for multiple comparisons
☐	☒	A full description of the statistical parameters including central tendency (e.g. means) or other basic estimates (e.g. regression coefficient) AND variation (e.g. standard deviation) or associated estimates of uncertainty (e.g. confidence intervals)
☐	☒	For null hypothesis testing, the test statistic (e.g. *F*, *t*, *r*) with confidence intervals, effect sizes, degrees of freedom and *P* value noted<br>*Give P values as exact values whenever suitable.*
☒	☐	For Bayesian analysis, information on the choice of priors and Markov chain Monte Carlo settings
☐	☒	For hierarchical and complex designs, identification of the appropriate level for tests and full reporting of outcomes
☐	☒	Estimates of effect sizes (e.g. Cohen's *d*, Pearson's *r*), indicating how they were calculated

Our web collection on statistics for biologists contains articles on many of the points above.

Software and code

Policy information about availability of computer code

Data collection	*No software was used for data collection.*
Data analysis	RNAseq reads were adapter-trimmed using Cutadapt (v1.14) and mapped to human-specific repetitive elements from RepBase (version 18.05) by STAR (v2.4.0i) (Dobin et al 2013). Repeat-mapping reads were removed, and remaining reads were mapped to the human genome assembly (hg38) with STAR. Read counts for all genes annotated in GENCODE v24 (hg38) were calculated using the read summarization program featureCounts (CDS regions only, Liao et al., 2014). Differential expression analysis between different experimental groups (HD untreated, HD Cas13d/NT, HD Cas13d/CAGEX, Control 1-3 untreated, Control 1-3 Cas13d/NT, Control 1-3 Cas13d/CAGEX was performed using DESeq2 (Love et al., 2014). Differentially expressed mRNAs were defined as having a false discovery rate of p <0.00001 unless otherwise specified.<br>Statistical analysis was performed with GraphPad Prism 9 software. The p-values less than 0.05 were considered as statistically significant.

For manuscripts utilizing custom algorithms or software that are central to the research but not yet described in published literature, software must be made available to editors and reviewers. We strongly encourage code deposition in a community repository (e.g. GitHub). See the Nature Portfolio guidelines for submitting code & software for further information.

Data

Policy information about availability of data

All manuscripts must include a data availability statement. This statement should provide the following information, where applicable:

- Accession codes, unique identifiers, or web links for publicly available datasets
- A description of any restrictions on data availability
- For clinical datasets or third party data, please ensure that the statement adheres to our policy

All RNAseq data discussed in this publication have been deposited in NCBI's Gene Expression Omnibus, accession number GSE214110 (https://www.ncbi.nlm.nih.gov/geo/query/acc.cgi?acc=GSE214110). There are no restrictions on data availability.

Field-specific reporting

Please select the one below that is the best fit for your research. If you are not sure, read the appropriate sections before making your selection.

☒ Life sciences ☐ Behavioural & social sciences ☐ Ecological, evolutionary & environmental sciences

For a reference copy of the document with all sections, see nature.com/documents/nr-reporting-summary-flat.pdf

Life sciences study design

All studies must disclose on these points even when the disclosure is negative.

Sample size	Based on means and standard deviations from previous studies with Q175/+ mice (69), a power analysis revealed that <3 mice reliably distinguish mutant and control populations (p<0.05). To have 95% confidence in detecting an improvement with inhibition of the mutant HTT allele, 4 animals per genotype and treatment group are required (power analysis: 2-way ANOVA, genotype x treatment, p<=0.05). No statistical methods were used to pre-determine sample sizes in our in vitro experiemnts. Sample size for experiments involving HEK293 cells were based from a previous study (Batra, et al 2017, 30). Sample size for experiments involving iPSC-derived neurons were based from Smith-Geater, 34). Data distribution was assumed to be normal but this was not formally tested.
Data exclusions	No data was excluded.
Replication	All replications were successful and included in our outcome measures. Technical replication is represented by N in the figure legends.
Randomization	Mice were randomized into groups to avoid bias. Balanced gender ratio was considered in all experiments. Randomization was not available for iPSC due to limited availability of patient lines and established neurotypical controls.
Blinding	Data were collected using animal or cell line ID and analyzed by investigators who were blinded to genotype and treatment.

Reporting for specific materials, systems and methods

We require information from authors about some types of materials, experimental systems and methods used in many studies. Here, indicate whether each material, system or method listed is relevant to your study. If you are not sure if a list item applies to your research, read the appropriate section before selecting a response.

Materials & experimental systems

n/a	Involved in the study	
☐	☒	Antibodies
☐	☒	Eukaryotic cell lines
☒	☐	Palaeontology and archaeology
☐	☒	Animals and other organisms
☒	☐	Human research participants
☒	☐	Clinical data
☒	☐	Dual use research of concern

Methods

n/a	Involved in the study	
☒	☐	ChIP-seq
☒	☐	Flow cytometry
☒	☐	MRI-based neuroimaging

Antibodies

Antibodies used	Primary: DARPP-32, 1:500, Abcam ab40801 (EP720Y); CTIP2, 1:500, Abcam ab18465 (25B6); anti-HTT, 1:1000, Millipore Sigma MAB 2166 (1HU-4C8); anti-poly-Q, 1:1000, Millipore Sigma MABN2427 (MW1); and β-actin, 1:5000, Sigma-Aldrich A5316 (AC-74); Anti-Huntingtin Protein Antibody 1:200, Merck Milipore MAB5347 (mEM48); Anti-polyQ Disease Proteins Antibody, 1:1000, MiliporeSigma MAB1574 (5TF1-1C2); anti-beta Tubulin, 1:5000, Abcam ab6046.Secondary: Alexa-fluor rabbit 555 IgG (A-31572, Invitrogen), rat 488 IgG (A48262, Invitrogen),mouse 555 igG (A32727, Invitrogen) and mouse 488 igG (A32723, Invitrogen);Anti-Rabbit IgG (Goat), HRP-Labeled (NEF812001, PerkinElmer); Anti-Mouse IgG (Goat), HRP-Labeled (NEF822001EA, PerkinElmer).
Validation	Antibody specificity was validated in knockout cell lines by the manufacturer prior to purchase for all antibodies used in this study. For antibodies used for western blot analysis (anti-HTT, 1:1000, Millipore Sigma MAB 2166 (1HU-4C8); anti-poly-Q, 1:1000, Millipore Sigma MABN2427 (MW1); and β-actin, 1:5000, Sigma-Aldrich A5316 (AC-74); Anti-Huntingtin Protein Antibody 1:200, Merck Milipore MAB5347 (mEM48); Anti-polyQ Disease Proteins Antibody, 1:1000, MiliporeSigma MAB1574 (5TF1-1C2); anti-beta Tubulin, 1:5000, Abcam ab6046), an antibody was considered validated if it produced a band (or bands) of the expected molecular weight(s) for the target protein. Both positive (primary antibody only) and negative controls (secondary antibody only) were used in immunocytochemistry assays to confirm proper protein labeling (DARPP-32, 1:500, Abcam ab40801 (EP720Y); CTIP2, 1:500, Abcam ab18465 (25B6);. Anti-Huntingtin Protein Antibody 1:200, Merck Milipore MAB5347 (mEM48).

Eukaryotic cell lines

Policy information about cell lines

Cell line source(s)

. HD stem cell lines 66, 77, and 109 were a gift from Dr. Leslie Thompson's lab at UC Irvine whereas control lines were a gift from the Goldstein lab at UCSD. HEK293Ts were obtained from Millipore Sigma (12022001). HD human fibroblast lines GM04723 (CAG15/67), GM02151 (CAG18/46) were obtained from the Coriell Cell Repository.

Authentication

Human iPSC lines derived from individuals with HD and neurotypical controls have been previously characterized elsewhere (Gore, et al; 2011, Smith-Geater et al 2020). Cells were routinely checked by karyotype and CNV arrays to avoid genomic alterations in the culture.

Mycoplasma contamination

All iPSC and striatal neuron cultures were routinely tested for mycoplasma by PCR. Media supernatants (with no antibiotics) were collected, centrifuged, and resuspended in saline buffer. Ten microliters of each sample were used for a PCR with the following primers: Forward: GGCGAATGGGTGAGTAAC; Reverse: CGGATAACGCTTGCGACCT. Only negative samples were used in the study.

Commonly misidentified lines
(See ICLAC register)

No commonly misidentified lines were used in this study.

Animals and other organisms

Policy information about studies involving animals; ARRIVE guidelines recommended for reporting animal research

Laboratory animals

Heterozygous zQ175 (zQ175/+) mice and wild type littermates (both gender- and age-matched in each group) were used in the study. zQ175 line in C57BL/6 background strain was obtained from The Jackson Laboratory (Bar Harbor, ME) and bred and maintained in our lab. All mice were housed under specific pathogen-free conditions with a reversed 12-h light/dark cycle maintained at 23˚C with 30% to 70% humidity and provided with food and water ad libitum. Data from different genders were analyzed grouped when there was no gender-dependent difference in the outcome measures.

Wild animals

This study did not involve wild animals.

Field-collected samples

No field-collected samples were used in this study.

Ethics oversight

The study was carried out in strict accordance with the recommendations in the Guide for the Care and Use of Laboratory Animals of the National Institutes of Health and approved by Animal Care and Use Committee at Johns Hopkins University.

Note that full information on the approval of the study protocol must also be provided in the manuscript.
