# Supplementary material for: An RNA-targeting CRISPR–Cas13d system alleviates disease-related phenotypes in Huntington’s disease models
Source: Nat Neurosci. 2022 Dec 12;26(1):27–38. doi: 10.1038/s41593-022-01207-1 (PMC9829537; doi:10.1038/s41593-022-01207-1)
Supplement: Source Data Fig. 5 — Unprocessed western blots. [file 41593_2022_1207_MOESM19_ESM.pdf]

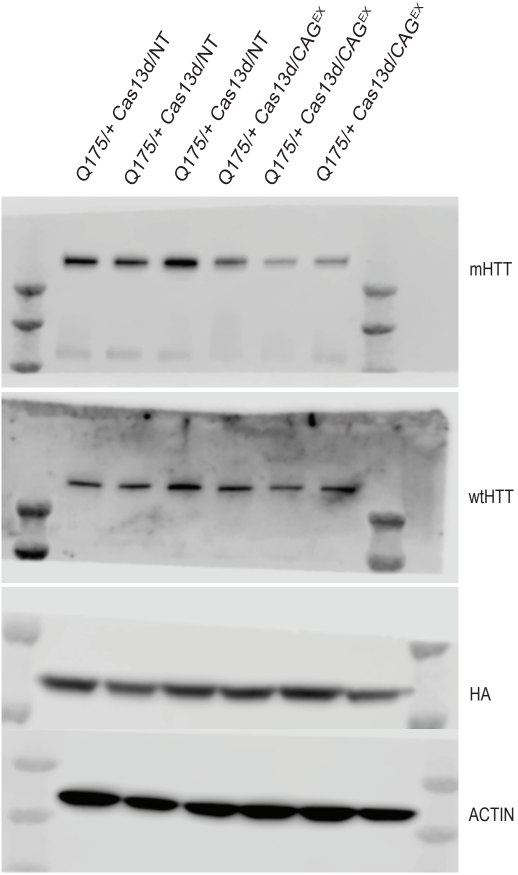
Q175/+ Cas13d/NT
Q175/+ Cas13d/NT
Q175/+ Cas13d/NT
Q175/+ Cas13d/CAG^EX
Q175/+ Cas13d/CAG^EX
Q175/+ Cas13d/CAG^EX
mHTT
wtHTT
HA
ACTIN

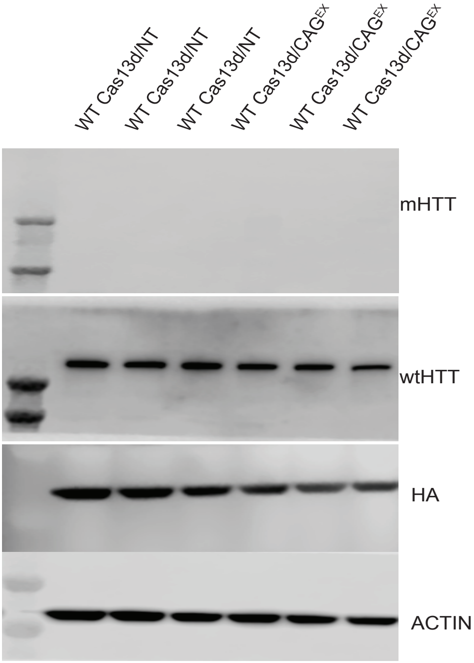

WT Cas13d/NT
WT Cas13d/NT
WT Cas13d/NT
WT Cas13d/CAG^EX
WT Cas13d/CAG^EX
WT Cas13d/CAG^EX
mHTT
wtHTT
HA
ACTIN
